# Allergic Asthma Favors *Brucella* Growth in the Lungs of Infected Mice

**DOI:** 10.3389/fimmu.2018.01856

**Published:** 2018-08-10

**Authors:** Arnaud Machelart, Georges Potemberg, Laurye Van Maele, Aurore Demars, Maxime Lagneaux, Carl De Trez, Catherine Sabatel, Fabrice Bureau, Sofie De Prins, Pauline Percier, Olivier Denis, Fabienne Jurion, Marta Romano, Jean-Marie Vanderwinden, Jean-Jacques Letesson, Eric Muraille

**Affiliations:** ^1^Unité de Recherche en Biologie des Microorganismes, Laboratoire d’Immunologie et de Microbiologie, NAmur Research Institute for Life Sciences (NARILIS), Université de Namur, Namur, Belgium; ^2^Institute for Medical Immunology, Université Libre de Bruxelles, Brussels, Belgium; ^3^Department of Molecular and Cellular Interactions, Vlaams Interuniversitair Instituut voor Biotechnologie, Vrije Universiteit Brussel, Brussels, Belgium; ^4^Laboratory of Cellular and Molecular Immunology, GIGA- Research & WELBIO, University of Liège, Liège, Belgium; ^5^Immunology Unit, Scientific Institute for Public Health (WIV-ISP), Brussels, Belgium; ^6^Laboratory of Neurophysiology, Université Libre de Bruxelles, Brussels, Belgium; ^7^Laboratoire de Parasitologie, Faculté de Médecine, Université Libre de Bruxelles, Brussels, Belgium

**Keywords:** allergic asthma, infection, *Brucella melitensis*, *Streptococcus pneumoniae*, brucellosis, *Mycobacterium tuberculosis*

## Abstract

Allergic asthma is a chronic Th2 inflammatory disease of the lower airways affecting a growing number of people worldwide. The impact of infections and microbiota composition on allergic asthma has been investigated frequently. Until now, however, there have been few attempts to investigate the impact of asthma on the control of infectious microorganisms and the underlying mechanisms. In this work, we characterize the consequences of allergic asthma on intranasal (i.n.) infection by *Brucella* bacteria in mice. We observed that i.n. sensitization with extracts of the house dust mite *Dermatophagoides farinae* or the mold *Alternaria alternata* (*Alt*) significantly increased the number of *Brucella melitensis, Brucella suis*, and *Brucella abortus* in the lungs of infected mice. Microscopic analysis showed dense aggregates of infected cells composed mainly of alveolar macrophages (CD11c^+^ F4/80^+^ MHCII^+^) surrounded by neutrophils (Ly-6G^+^). Asthma-induced *Brucella* susceptibility appears to be dependent on CD4^+^ T cells, the IL-4/STAT6 signaling pathway and IL-10, and is maintained in IL-12- and IFN-γR-deficient mice. The effects of the *Alt* sensitization protocol were also tested on *Streptococcus pneumoniae* and *Mycobacterium tuberculosis* pulmonary infections. Surprisingly, we observed that *Alt* sensitization strongly increases the survival of *S. pneumoniae* infected mice by a T cell and STAT6 independent signaling pathway. In contrast, the course of *M. tuberculosis* infection is not affected in the lungs of sensitized mice. Our work demonstrates that the impact of the same allergic sensitization protocol can be neutral, negative, or positive with regard to the resistance of mice to bacterial infection, depending on the bacterial species.

## Introduction

One striking feature of infectious diseases is the marked interindividual variation in susceptibility/resistance. Interestingly, individuals with the highest level of infection are also often the major disseminators (termed super-spreaders) of an epidemic among a population (1). Thus, with a better understanding of the factors predisposing individuals to susceptibility to a particular infectious agent, we may be able to both anticipate and better treat individual infections and more efficiently control the dissemination of infection among populations.

Allergic asthma is one of the most common lung diseases. It affects an estimated 300 million people worldwide (2) and its prevalence continues to increase in many parts of the world (3). It is characterized by recurring symptoms of reversible airflow obstruction, bronchial hyperresponsiveness and lower airway inflammation. The lungs of atopic individuals display increased levels of IL-4-mediated (Th2) inflammation and eosinophilia (4). The impact of infections and host microbiota composition on allergic asthma has been investigated frequently [reviewed in Ref. (5)]. However, despite the significant proportion of people worldwide affected by asthma and though some epidemiological studies (6–8) report increasingly severe bacterial and viral infections in asthma patients, little effort has been made to investigate the impact of asthma on the control of infectious microorganisms and identify the underlying mechanisms.

*Brucella* (alpha-proteobacteria) are facultative intracellular Gram-negative coccobacilli that infect wild and domestic mammals and cause brucellosis. Human brucellosis is a zoonotic infection transmitted mainly through ingestion and inhalation (9). Airborne transmission is a major cause of outbreaks of human brucellosis in bovine and porcine slaughterhouses, vaccine production laboratories, research laboratories, and rural areas (10–13). Due to its easy aerosolization, high infectivity, and airborne transmission, *Brucella* species are considered potential biological weapons (14, 15) and are classified as category B bioterrorism agents. Four species of *Brucella* can cause human disease: *Brucella melitensis, Brucella abortus, Brucella suis*, and *Brucella canis*. The vast majority of cases worldwide are attributed to *B. melitensis* [reviewed in Ref. (16)]. Although it is rarely fatal, *Brucella* can cause a devastating multi-organ disease in humans with serious health complications in the absence of prolonged antibiotic treatment (16, 17). Despite significant progress, the incidence of human brucellosis remains very high in endemic areas (18) and is considered to be largely underestimated (19, 20). Currently, there are no effective vaccines available to protect humans. All commercially available animal vaccines are live vaccines that would cause disease in humans (21, 22). In a mouse intranasal (i.n.) infection model, we demonstrated previously that IFN-γ-producing CD4^+^ T cells (Th1) are key actors in the control of *Brucella* multiplication and persistence in lungs (23).

In the current study, we developed an original experimental model to analyze the impact of allergic asthma sensitization on the course of i.n. *Brucella* infection in mice. Extracts of the house dust mite *Dermatophagoides farinae* (HDM) (24) and the mold *Alternaria alternata* (*Alt*) (25), which are both recognized as important causes of respiratory allergies, were used to sensitize mice before and during infection. Our results showed that allergic asthma increases the susceptibility of mice to *Brucella* infection. However, this phenomenon cannot be generalized to other bacterial infections as we observed that the same protocol led to better control of *Streptococcus pneumoniae* and had no effect on the course of *Mycobacterium tuberculosis* infections in a murine model.

## Materials and Methods

### Ethics Statement

The procedures used in this study and the handling of the mice complied with current European legislation (directive 86/609/EEC) and the corresponding Belgian law “Arrêté royal relatif à la protection des animaux d’expérience du 6 avril 2010 publié le 14 mai 2010.” The Animal Welfare Committee of the Université de Namur (UNamur, Belgium) reviewed and approved the complete protocol for *Brucella* infections (Permit Number: UN-LE-14/220). The Animal Welfare Committee of the Université Libre de Bruxelles (ULB, Belgium) reviewed and approved the complete protocol for *S. pneumoniae* infections (Permit Number: ULB-IBMM-2016-21-88). The Ethics committee of the WIV-ISP and CODA-CERVA approved the complete protocol for *M. tuberculosis* infections (ethics agreement number 201405-14-01).

### Mice and Reagents

Wild-type BALB/c and C57BL/6 mice were acquired from Harlan (Bicester, UK). STAT6^−/−^ BALB/c mice, IL4^−/−^ BALB/c mice, IL12p40^−/−^ BALB/c mice, TCR-δ^−/−^ C57BL/6 and IL-10GFP transgenic [B6(Cg)-Il10tm1.1Karp/J] C57BL/6 mice were all purchased from The Jackson Laboratory (Bar Harbor, ME, USA). IFN-γR^−/−^ (26) and IL-12p35^−/−^ C57BL/6 mice (27) were acquired from Dr. B. Ryffel (University of Orleans, France). TAP1^−/−^ C57BL/6 mice (28), MHCII^−/−^ C57BL/6 mice (29) were acquired from Jörg Reimann (University of Ulm, Ulm, Germany). IL-10^−/−^ BALB/c mice were obtained from Guillaume Holdenhove (Université Libre de Bruxelles, Gosselies, Belgium). All wild-type and deficient mice used in this study were bred in the animal facility of the Gosselies campus of the Université Libre de Bruxelles (ULB, Belgium). We used wild-type strains of *B. melitensis* 16M and *B. suis* 1330. We also used *B. melitensis* 16M and *B. abortus* 2308 strains stably expressing a rapidly maturing variant of the red fluorescent protein DsRed (30), the mCherry protein (mCherry-*Br*), under the control of the strong *Brucella* spp. promoter, PsojA. Construction of the mCherry-*Br* strains has been described previously in detail (31). All *Brucella* were handled under BSL-3 containment according to Council Directive 98/81/EC of 26 October 1998 and a law of the Walloon government of 4 July 2002.

Cultures were grown overnight with shaking at 37°C in 2YT medium (Luria-Bertani brothwith double quantity of yeast extract) and were washed twice in RPMI 1640 (Gibco Laboratories) (3,500 × *g*, 10 min) before inoculation of the mice.

### Allergens and Allergic Asthma Sensitization Protocol

Lyophilized house dust mite *D. farinae* (abbreviated HDM) extracts were from Greer Laboratories (Lenoir, NC, USA). *A. alternata* (strain18586) (abbreviated *Alt*) was obtained from the BCCM™/IHEM (Institute of Public Health, WIV-ISP, Brussels, Belgium) and cultured for 3 weeks at 27°C in flasks containing 250 ml of Czapek medium. Mold pellicles were harvested and homogenized in 0.4% NH_4_HCO_3_ + polyvinyl polypyrrolidone (Sigma) with an ultra-thurax. The homogenates were then shaken for 3 h at 4°C. Extracts were centrifuged twice for 30 min at 20,000 *g*, dialyzed against phosphate-buffered saline (PBS) and stored at −20°C in 50% glycerol.

For asthma sensitization, mice were lightly anesthetized with isoflurane [from Abbott laboratories (# No. B506)]. Once the mice were unresponsive but breathing comfortably, a solution of HDM (100 µg of *D. farinae* extract in 50 µl of PBS) or *Alt* (5 µg of *A. alternata* extract in 100 µl of PBS) was applied directly to the nostrils. The animals were allowed to slowly inhale the liquid and were then allowed to recover in a supine position. For the HDM model, mice were instilled intranasally (i.n.) once per week throughout the experiment (32) and for the *Alt* model mice received the extract twice per week throughout the experiment (25). Mice were infected 17 days after the first instillation.

### *Brucella* Infection

Mice were anesthetized with a cocktail of Xylasine (9 mg/kg) and Ketamine (36 mg/kg) in PBS before being inoculated i.n. with 2 × 10^3^ or 2 × 10^4^ CFU of *B. melitensis*, as indicated, 2 × 10^3^ CFU of *B. abortus*, or 2 × 10^3^ CFU of *B. suis*. We used wild-type or mCherry-expressing *Brucella* in 30 µl of PBS [described in Ref. (31)]. Control animals were inoculated with the same volume of PBS. The infectious doses were validated by plating serial dilutions of the inoculums. At the selected time after infection, mice were sacrificed by cervical dislocation. Immediately after sacrifice, spleen, liver, and lung cells were collected for bacterial count, flow cytometry, and/or microscopic analyses. All infections were performed in an Animal Biosafety Level 3 facility.

For bacterial counting, organs were crushed and transferred to PBS/0.1% X-100 Triton (Sigma-Aldrich). We performed successive serial dilutions in RPMI to obtain the most accurate bacterial count and plated them on 2YT medium. The CFU were counted after 5 days of culture at 37°C.

### *S. pneumoniae* Infection

*Streptococcus pneumoniae* serotype 1 (clinical isolate E1586) were grown in Todd Hewitt Yeast Broth (Sigma-Aldrich) as described previously (33). For infection, frozen working stocks were washed and diluted in PBS. Mice were anesthetized by intraperitoneal injection of ketamine–xylazine and 20 µl of the lethal inoculum (2 × 10^7^ CFU) were administered by i.n. route. Mouse survival was recorded every 24 h.

### *M. tuberculosis* Infection

BALB/c mice were infected with 50–100 CFU of virulent *M. tuberculosis* H37Rv using a nose-only inhalation exposure system (CH Technologies, Inc. Westwood, NJ, USA). The *M. tuberculosis* H37Rv strain used was grown for 2 weeks as a surface pellicle on Sauton medium and stored frozen in aliquots at −80°C. For bacterial counting, serial threefold total lung homogenate dilutions were plated on 7H11 Middlebrook agar supplemented with oleic acid-albumin-dextrose-catalase. Colonies were counted visually after 4 weeks. CFU counts obtained from two or three dilutions were used to calculate the total number of CFU/lung/mouse. Data are expressed as log10 CFU per organ per mouse. All *M. tuberculosis* infections were performed in a BSL3 facility at the Scientific Institute of Public Health (WIV-ISP) according to rules established by the ethics committee of the WIV-ISP and CODA-CERVA (ethics agreement number 201405-14-01).

### Bronchoalveolar Lavages (BALs)

Mice were euthanized by cervical dislocation. The trachea was exposed and incised. A needle (1.2 mm × 40 mm) was inserted into the trachea and bronchoalveolar fluid was harvested by washing the lungs twice with 1 ml of PBS. Total cell counts were determined with a hemacytometer. Differential cell counts were obtained by counting at least 500 cells on cytospin slides stained with Diff-Quick (Dade Behring).

### Determination of Serum Levels of Total IgE

Serum IgE levels in sera were determined using a sandwich ELISA. Plates were coated with a rat antimouse IgE mAb (LO-ME-2, IMEX, UCL, Brussels, Belgium) and saturated. Serial twofold dilutions of serum or purified monoclonal mouse IgE (LB-4, IMEX, UCL, Brussels, Belgium) were applied for 2 h. Then, peroxidase labeled rat antimouse IgE (LO-ME-3) was used. After 1 h of incubation at room temperature (RT), plates were washed four times in PBS, and 100 µl substrate solution (BD OptEiA; BD Biosciences) was added to each well. After 10 min of incubation at RT in the dark, the enzyme reaction was stopped by adding 25 μl/well 2 N H_2_SO_4_, and absorbance was measured at 450 nm.

### Quantitative PCR for IL-4

Total RNA was extracted from homogenized lung cells with Tri-reagent (Sigma-Aldrich), according to the manufacturer’s instructions 24 h after the last instillation. cDNA was synthesized using the Promega GoScript Reverse Transcription System. RT-qPCR was performed on a Stratagene Mx 3000P using a qPCR Go Taq master mix with Bryt Green (Promega). Each RT-qPCR amplification was performed in duplicate under the following conditions: 95°C for 10 min, followed by 40 cycles at 95°C for 15 s and 60°C for 1 min. The forward and reverse primers used for IL-4 were 5-GTGCAGCTTATCGATGAATCC-3′ and 5-AGCCATATCCACGGATGCGAC-3, Hydroxymethylbilane synthase (HmBS, forward 5′-GAAACTCTGCTTCGCTGCATT-3′, reverse 5′-TGCCCATCTTTCATCACTGTATG-3′) mRNA was used as the reference housekeeping gene for normalization. For each sample (*x*), the normalization factor was calculated using the formula, mean of Ct of ref(*i*) − Ct of ref(*x*), where *i* represents all samples (Ct is on the threshold cycle-value shown as the mean of three different RT-qPCR reactions), as described in Ref. (34). The level of target mRNA, relative to the mean of the reference housekeeping gene, was calculated by raising 2 to the power of {40 − [Ct of target + mean of (Ct of norm. HmBS)]}.

### Determination of Arginase Activity

Arginase activity was determined by measuring the total production of urea from lung homogenates. Individual lungs were crushed and transferred to 1 ml of PBS. The homogenates were centrifuged, and urea levels were determined in the supernatant using an Arginase Activity Assay kit (Sigma MAK112). Arginase activity is expressed as units per liter of sample, where 1 unit of arginase converts 1 μmole of l-arginine to ornithine and urea per min at pH 9.5 and 37°C.

### Arginase Inhibition

Mice were injected i.n. with 200 µg of nor-NOHA (N-hydroxy-nor-l-arginine, Enzolifesciences) in 50 µl of PBS on days 8, 15, 18, 21, 24, and 27 post first mold instillation. In order to verify the effectiveness of the inhibitor, we measured the arginase activity using the Arginase Activity Assay kit (Sigma MAK112) on naive and asthmatic lung extracts in the presence or absence of nor-NOHA (2 mg/ml) during the arginase reaction.

### Cytofluorometric Analysis

As described previously (23), lungs were harvested, cut into small pieces, and incubated for 1 h at 37°C with a mix of DNAse I fraction IX (Sigma-Aldrich) (100 µg/ml) and 1.6 mg/ml of collagenase (400 Mandl U/ml). Lung cells were washed and filtered, and then incubated with saturating doses of purified 2.4G2 (anti-mouse Fc receptor, ATCC) in 200 µl PBS, 0.2% BSA, 0.02% NaN3 (FACS buffer) for 20 min at 4°C to prevent antibody (Ab) binding to the Fc receptor. Various fluorescent mAb combinations in FACS buffer were used to stain 3–5 × 10^6^ cells. We acquired the following mAbs from BD Biosciences: Fluorescein (FITC)-coupled HL3 (anti-CD11c), FITC-coupled 145-2C11 (anti-CD3ε), FITC-coupled M1/70 (anti-CD11b), FITC-coupled 1A8 (anti-Ly6G), phycoerythrin (PE)-coupled RM4-5 (anti-CD4), PE-coupled 53-6.7 (anti-CD8α), PE-coupled PK136 (anti-NK1.1), PE-coupled GL3 (anti–TCR-γ/δ), PE-coupled E50-2440 (anti-SIGLEC-F), allophycocyanin (APC)-coupled BM8 (anti-F4/80), biotin-coupled M5/114.15.2 (anti-MHCII, I-A/I-E), and biotin-coupled 307707 (anti-CD101). Incubation with a streptavidin-coupled APC for 30 min was necessary for the biotin-coupled Ab. The cells were analyzed on a FACScalibur cytofluorometer. Dead cells and debris were eliminated from the analysis according to size and scatter.

### Immunofluorescence Microscopy

Lungs were fixed for 2 h at RT in 2% paraformaldehyde (pH 7.4), washed in PBS, and incubated overnight at RT in a 20% PBS-sucrose solution under a vacuum. Tissues were then embedded in the Tissue-Tek OCT compound (Sakura), frozen in liquid nitrogen, and cryostat sections (5 µm) were prepared. For staining, tissue sections were rehydrated in PBS and incubated in a PBS solution containing 1% blocking reagent (Boeringer) (PBS-BR 1%) for 20 min before incubation overnight in PBS-BR 1% containing any of the following mAbs or reagents: DAPI nucleic acid stain Alexa Fluor 350, 488 phalloidin (Molecular Probes), biotin-coupled 1A8 (anti-Ly6G), biotin-coupled 53-2.1 (anti-CD90.2), biotin-coupled polyclonal anti mouse Major Binding Protein (MBP, MyBioSource), Alexa Fluor^®^ 647-coupled M5/114.15.2 (anti-I-A/I-E, MHCII), APC-coupled BM8 (anti-F4/80, Abcam), Alexa Fluor 647-coupled HL3 (anti-CD11c, BD Biosciences). Incubation with a streptavidin-coupled APC for 2 h was necessary for the biotin-coupled Ab. iNOS and arginase-1 were detected using IgG H-52 (anti-Arg1, Santa Cruz Biotechnology) and rabbit polyclonal anti-NOS2 Ab (Calbiochem), respectively and were stained with a secondary Ab, Alexa Fluor 647-coupled goat anti-rabbit IgG (Molecular Probes). Slides were mounted in Fluoro-Gel medium (Electron Microscopy Sciences, Hatfield, PA, USA). Labeled tissue sections were visualized with an Axiovert M200 inverted microscope (Zeiss, Iena, Germany) equipped with a high-resolution monochrome camera (AxioCam HR, Zeiss). Images (1,384 × 1,036 pixels, 0.16 μm/pixel) were acquired sequentially for each fluorochrome with A-Plan 10×/0.25 N.A. and LD-Plan-NeoFluar 63×/0.75 N.A. dry objectives and recorded as eight-bit gray-level *.zvi files. At least three slides were analyzed per organ from three different animals and the results are representative of two independent experiments.

### Confocal Microscopy

Confocal analyses were performed using the LSM780 confocal system fitted on an Observer Z 1 inverted microscope equipped with an alpha Plan Apochromat 63×/1.46 NA oil immersion objective (Zeiss, Iena, Germany). Hoechst/DAPI was excited using a 405 nm blue diode, and emission was detected using a band-pass filter (410–480 nm). The 488 nm excitation wavelength of the Argon/2 laser was used in combination with a band-pass emission filter (BP500–535 nm) to detect Alexa Fluor 488 phalloidin. The 543 nm excitation wavelength of the HeNe1 laser, and a band-pass emission filter (BP580–640 nm) were used for the red fluorochrome mCherry. The 633 excitation wavelength of the HeNe2 laser, and a band-pass emission filter (BP660–695 nm) were used for far-red fluorochromes such as APC. To ensure optimal separation of the fluorochromes, blue and red signals were acquired simultaneously in one track and green and far-red signals were acquired in a second track. The electronic zoom factor and stack depth were adjusted to the region of interest while keeping image scaling constant (*x*–*y*: 0.066 µm, *z*: 0.287 µm). A line average of 4 was used and datasets were stored as 8-bit proprietary *.czi files. The images were displayed using Zen2012 software (Zeiss) with linear manual contrast adjustment and exported as 8-bit uncompressed *.TIF images. The Figures, representing single optical sections across the region of interest, were prepared using the Canvas program.

### Hematoxylin-Eosin-Safran (HES) Histology

Lungs were fixed in 10% formalin. After fixation overnight, the lungs were embedded in paraffin. Tissues were sliced and 5 µm sections were stained with HES for light microscopy examination of the lung inflammation.

### Statistical Analysis

We used a (Wilcoxon-)Mann–Whitney test provided by the GraphPad Prism software to statistically analyze our results. Each group of deficient mice was compared to the wild-type mice. We also compared each group with the others and displayed the results when required. Values of *p* < 0.05 were considered to represent a significant difference. *, **, *** denote *p* < 0.05, *p* < 0.01, *p* < 0.001, respectively.

## Results

### Allergic Asthma Favors the Multiplication of *Brucella* in the Lungs

Repeated intranasal (i.n.) sensitization with an extract from the house dust mite *D. farinae* (HDM) (24) or an extract of the mold *A. alternata* (*Alt*) (25) has been described to induce allergic asthma in murine models. Under our experimental conditions, BALB/c mice sensitized during 17 days according to standard protocols (see Materials and Methods) display readily detectable cellular infiltration in the lungs (Figure S1A in Supplementary Material). BALs showed an increased number of lymphocytes, macrophages, neutrophils, and eosinophils (Figure S1B in Supplementary Material). As expected, this phenomenon was associated with increased IL-4 mRNA expression in the lungs (Figure S1C in Supplementary Material) and increased total IgE in the blood (Figure S1D in Supplementary Material). *Alt* induced stronger cellular recruitment in the lungs, but closely similar IL-4 and IgE levels compared to HDM.

**Figure 1:**
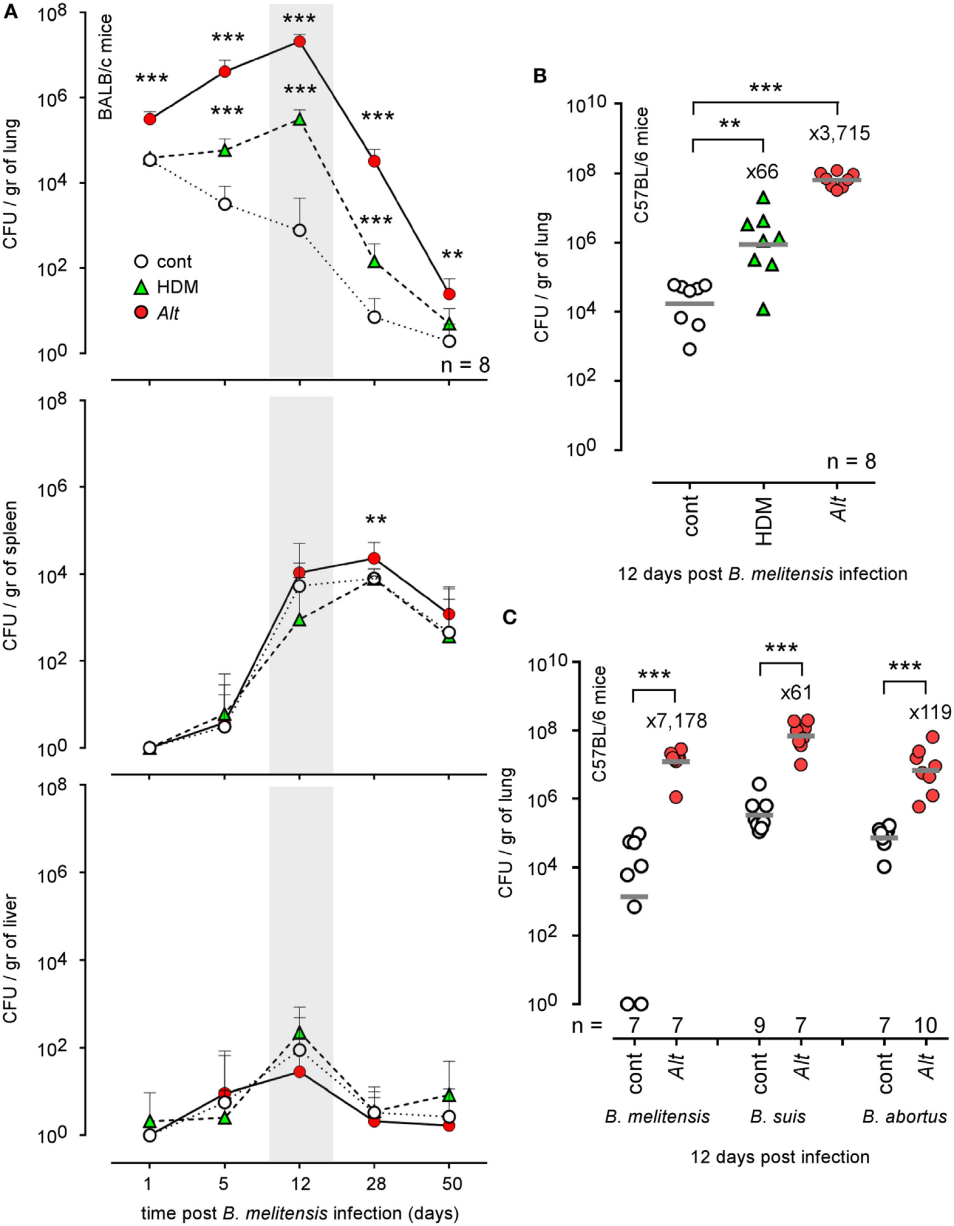
Impact of allergic asthma sensitization on the course of *Brucella* infection in wild-type mice. Wild-type BALB/c and C57BL/6 mice received repeated i.n. administration of phosphate-buffered saline, HDM, or *Alt* before i.n. infection with 2 × 10^4^ CFU of mCherry-*Brucella melitensis* **(A,B)** or 2 × 10^3^ CFU of *B. melitensis, B. abortus*, or *Brucella suis* **(C)**, as indicated in the figure and in the Section “Materials and Methods.” The mice were sacrificed at the selected time post infection. The data represent the number of CFU/gr of lung, spleen, and liver. *n* denotes the number of mice used for each lineage. These results are representative of at least two independent experiments. **p < 0.01, ***p < 0.001.

IFN-γ producing CD4^+^ T (Th1) cells play a key role in the control of *B. melitensis* infection (23). As chronic IL-4-dominated Th2 response induced by helminthes is well known to negatively affect the Th1 response (35–41), we compared the ability of groups of mice subjected to i.n. sensitization with PBS (cont), HDM, or *Alt* for 17 days to control i.n. *B. melitensis* infection. We chose a dose of *Brucella* infection similar to that used in our previous characterization of the *B. melitensis* intranasal infection model (23): 2 × 10^4^ CFU, as described in the Section “Materials and Methods.” This was a compromise to limit the individual variability resulting from the use of a low dose of infection (10^2^–10^3^ CFU) and the pulmonary inflammation resulting from a high dose of infection (10^6^–10^7^ CFU). It is important to note that the sensitization treatments were continued throughout the course of infection in all of our experiments to mimic chronic asthma. We observed that HDM and *Alt* sensitization negatively affect the control of *B. melitensis* infection in the lungs but have a weak or no effect in the spleen and liver (Figure 1A). Asthmatic mice display strongly enhanced bacterial loads in the lungs, with mean bacteria CFU/gr that were 416 times higher in the HDM group and 26,302 times higher in the *Alt* group compared to the control group at 12 days post infection.

Genetic background is important to the Th1/Th2 balance in mice. C57BL/6 mice preferentially mount a Th1 response with high IFN-γ and low IL-4, whereas that in BALB/c mice is a Th2 response with low IFN-γ and high IL-4 *in vitro* (42) and *in vivo* (43). Thus, C57BL/6 is regarded as a prototypic Th1-biased mouse strain. Despite this Th1 bias, C57BL/6 mice sensitized with HDM or *Alt* also display enhanced bacterial growth in the lungs (Figure 1B), demonstrating that this phenomenon is not restricted to Th2-biased BALB/c mice. Finally, to generalize our observation, we also tested the impact of *Alt* sensitization on the ability of BALB/c mice to control *B. suis* and *B. abortus* infection, the two other classical *Brucella* species causing human brucellosis. At 12 days post infection, we observed that asthmatic mice display enhanced levels of both *Brucella* strain in the lungs (Figure 1C). Note that we used a lower dose (2 × 10^3^ CFU) of *B. melitensis, B. suis*, and *B. abortus* to infect the mice, because *B. suis* and *B. abortus* already display a higher level of growth and persistence in the lungs of control mice compared to *B. melitensis*.

**Figure 2:**
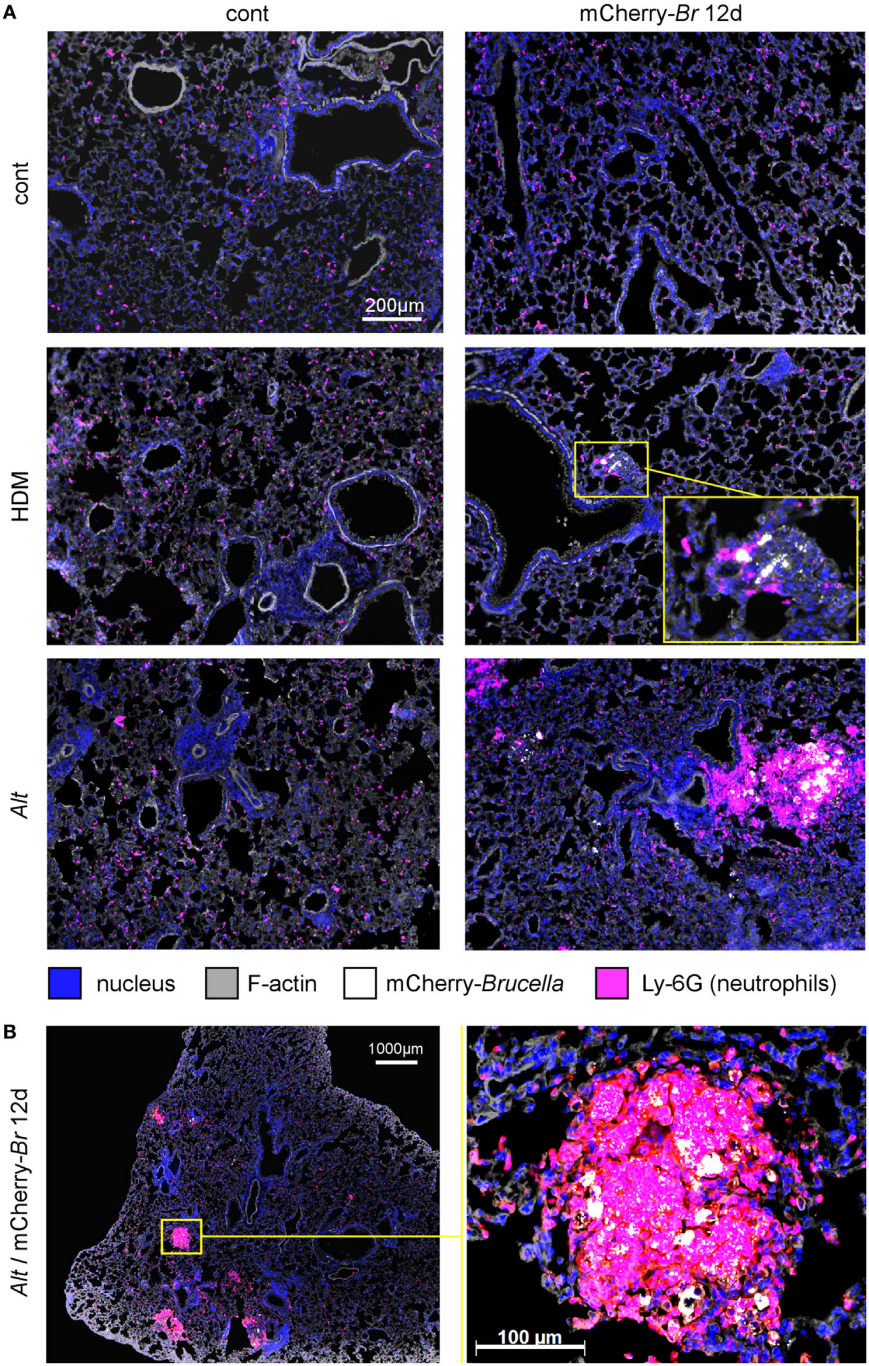
Microscopic analysis of lungs from control, HDM and *Alt* sensitized BALB/c mice. Wild-type BALB/c mice received repeated i.n. administration of phosphate-buffered saline (PBS) or *Alt* before i.n. inoculation of PBS or 2 × 10^4^ CFU of mCherry-*Brucella melitensis*. The mice were sacrificed at 12 days post infection. The lungs were harvested and fixed. Frozen sections were examined by immunohistofluorescence for bacteria (mCherry signal) and Ly-6G-expressing cells. **(A)** Comparison of control (cont), HDM, and *Alt* sensitized mice, infected or not. **(B)** High magnification representative view from *Alt* sensitized infected mice. The panels are color-coded by Ag as indicated. The data are representative of two independent experiments.

Using mCherry-expressing *B. melitensis* (31), we performed a microscopic fluorescent analysis to identify infected cells in lung tissues from control HDM- and *Alt*-sensitized BALB/c mice. Infected lungs from asthmatic mice display large and dense aggregates of Ly-6G^+^ cells, presumably neutrophils, surrounding highly infected cells (Figures 2A,B). These aggregates are not observed in the other groups of mice. Due to a low CFU level, infected cells could not be detected in the control mice.

**Figure 3:**
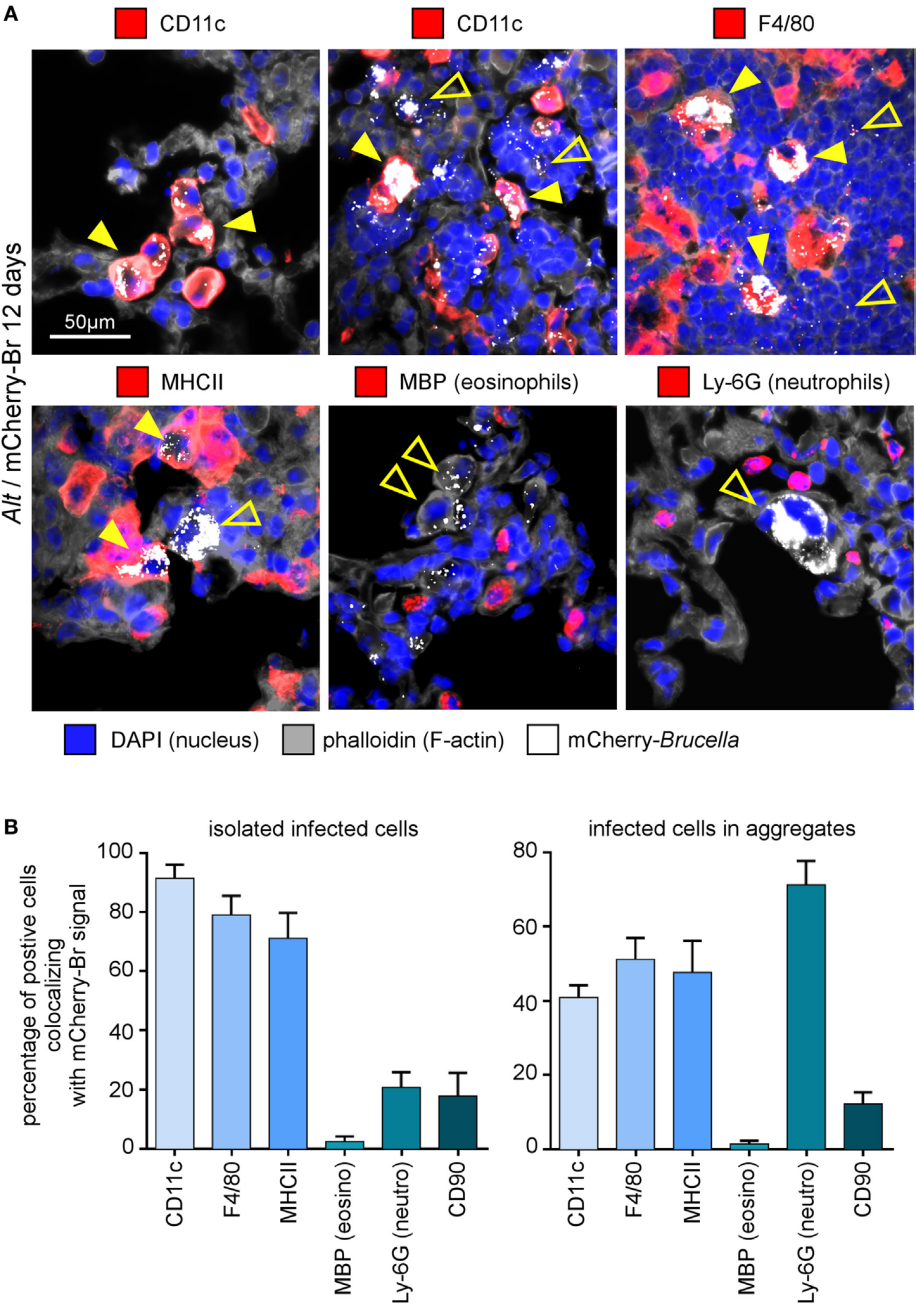
Cell surface phenotype of *Brucella melitensis* infected cells in asthmatic lungs. Wild-type BALB/c mice received repeated i.n. administration of *Alt* before i.n. infection with 2 × 10^4^ CFU of mCherry-*B. melitensis*. The mice were sacrificed at 12 days post infection. The lungs were harvested and fixed. Frozen sections were examined by immunohistofluorescence for mCherry (*Brucella*), CD11c, F4/80, MHCII, major basic protein (MBP), Ly-6G, and CD90 signals. The panels are color-coded by antigen as indicated. **(A)** High magnification representative view of infected cells in the lungs. **(B)** The data represent the percentage of mCherry-*Br* signal that co-localizes with CD11c, F4/80, MHCII, MBP, Ly-6G, and CD90 markers. When >12 infected cells are observed in the same observation field (approximately 700 μm × 500 μm), they are considered as “aggregated,” and when <12 are observed, they are considered as “isolated.” At least 200 infected cells from three different infected mice were analyzed for each staining. These data are taken from two independent experiments.

**Figure 4:**
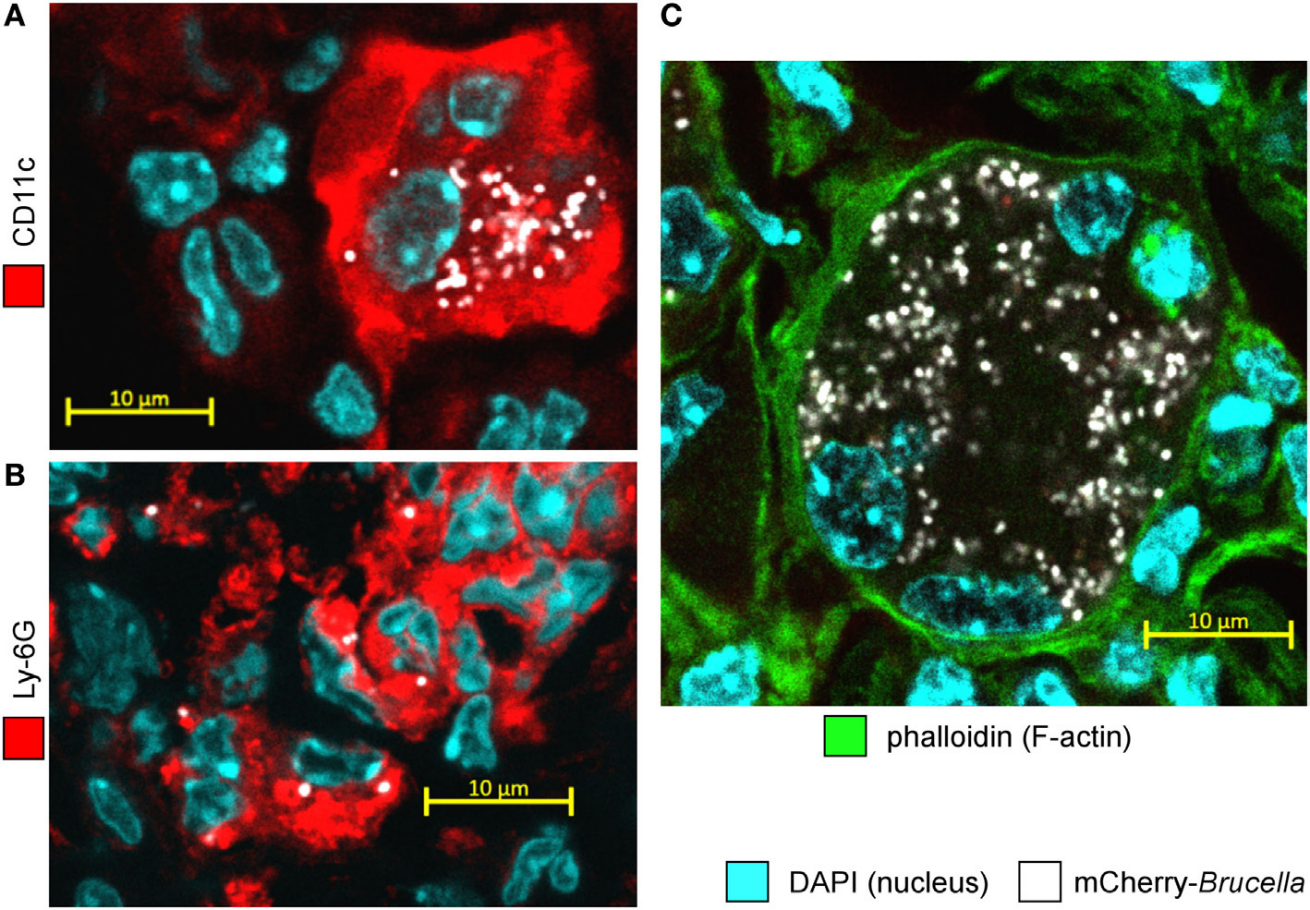
Alveolar macrophages and neutrophils are both infected with *Brucella melitensis* in the lungs of *Alt-*sensitized BALB/c mice. Wild-type BALB/c mice received repeated i.n. administration of *Alt* before i.n. infection with 2 × 10^4^ CFU of mCherry-*B. melitensis*. The mice were sacrificed at 12 days post infection. The lungs were harvested, fixed, and frozen sections were immunostained and examined by confocal microscopy. **(A,B)** Representative confocal images of single optical sections of mCherry-*Br* infected CD11c^+^ cells and Ly-6G^+^ cells stained with DAPI from *Alt* sensitized wild-type BALB/c mice. **(C)** Representative infected multinucleated giant cells stained with DAPI and phalloidin. The panels are color-coded for DAPI, phalloidin, the cell surface antigen examined or mCherry-*Brucella*. Scale bars = 10 µm, as indicated.

In asthmatic mice, mCherry^+^ infected cells are heterogeneous in phenotype and morphology (Figure 3A). A large fraction of the mCherry signal colocalizes with CD11c, F4/80, MHCII, and Ly-6G markers but rarely with major basic protein (MBP) or CD90 (Figure 3B), suggesting that infected cells are predominantly composed of alveolar macrophages (CD11c^+^ F4/80^+^ MHCII^+^) and neutrophils (Ly-6G^+^), but not of eosinophils (MBP^+^) or T cells (CD90^+^). The more highly infected cells are large cells with a typical alveolar macrophage morphology and colocalizing with the CD11c, F4/80, and MHCII staining. In contrast, the infected cells are small cells displaying fewer bacteria and are mainly observed in dense aggregates surrounding highly infected alveolar macrophages (Figure 2B). Infection of the Ly-6G*^+^* neutrophils (Figure 4A) and CD11c*^+^* alveolar macrophages (Figure 4B) was confirmed by confocal microscopy. We also observed the presence of highly infected multinucleated giant cells in the infected asthmatic mice (Figure 4C). These cells express low levels of CD11c, suggesting that they could result from the fusion of infected alveolar macrophages.

### Allergic Asthma-Induced Susceptibility to *Brucella* Is Dependent on the IL-4/STAT6 Signaling Pathway and CD4^+^ T Cells

**Figure 5:**
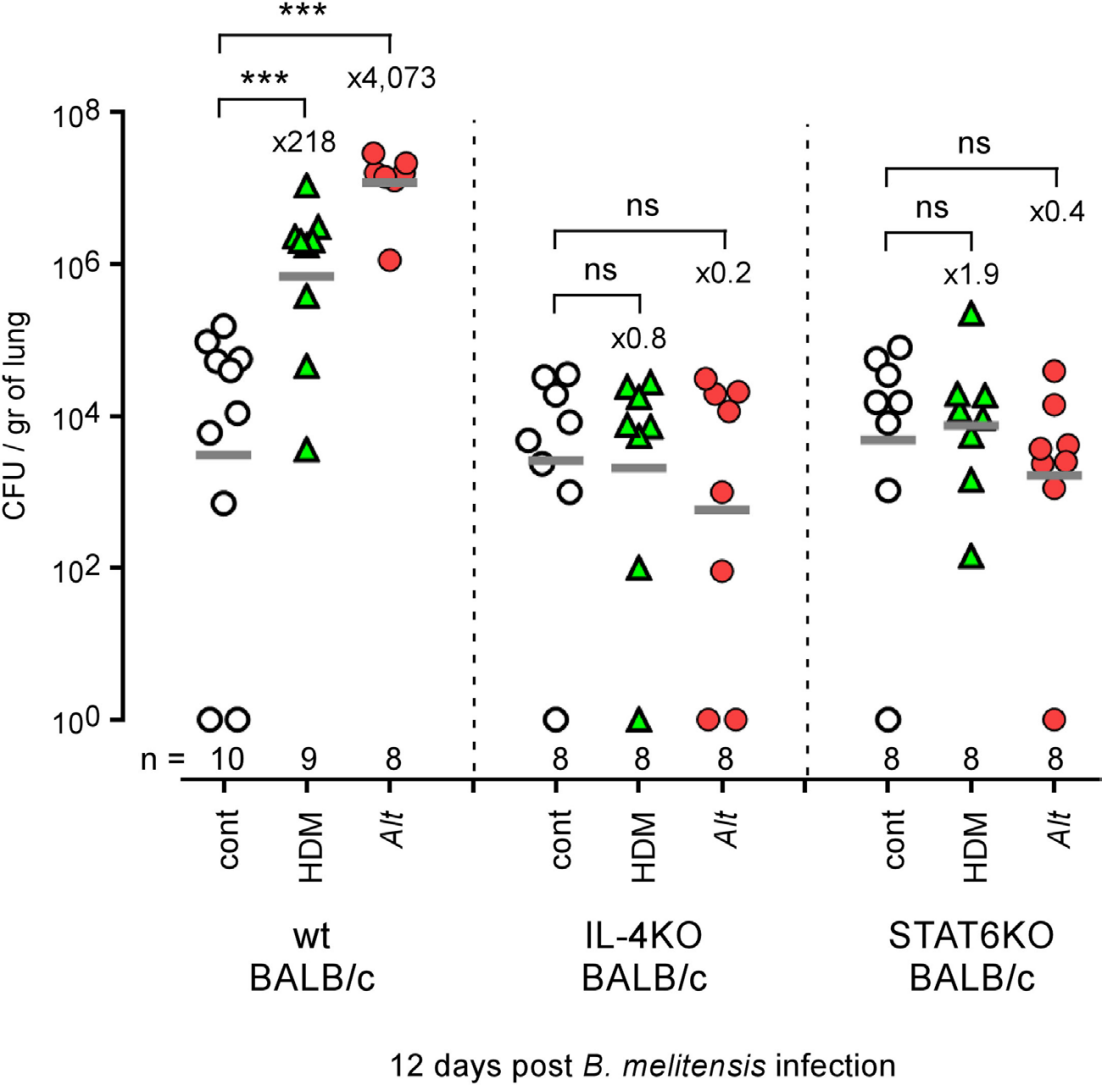
Impact of IL-4 and STAT6 deficiency on asthma-induced *Brucella* susceptibility in BALB/c mice. Wild-type, IL4^−/−^ and STAT6^−/−^ BALB/c mice received repeated i.n. administration of phosphate-buffered saline, HDM, or *Alt* before i.n. infection with 2 × 10^4^ CFU of mCherry-*Brucella melitensis*. The mice were sacrificed at 12 days post infection. The data represent the number of CFU/gr of lung at 12 days post infection from each group. *n* denotes the number of mice used for each lineage. These results are representative of at least two independent experiments. ***p < 0.001.

Mice deficient for IL-4, IL-13, or STAT6 develop attenuation of certain features of asthma, including eosinophil recruitment and airway hyperresponsiveness (44, 45). However, DNA microarray profile analysis of wild-type and STAT6^−/−^ asthmatic mice showed that a large portion of the transcriptional asthma signature is STAT6 independent (46). Comparative analysis of wild-type and STAT6^−/−^ asthmatic lungs showed that sensitized STAT6^−/−^ mice displayed reduced IgE levels in the blood (Figure S2A in Supplementary Material) and reduced infiltration of lymphocytes and eosinophils in the lung airways (Figure S2B in Supplementary Material). In contrast, macrophage and neutrophil infiltration remains much higher in sensitized STAT6^−/−^ mice than in control STAT6^−/−^ mice. As neutrophils have been reported to exert a suppressive effect on the protective response against *Brucella* (47), we thought first to determine whether enhanced susceptibility to *B. melitensis* lung infection induced by HDM and *Alt* treatment is dependent or independent of the IL-4/STAT6 signaling pathway. We, therefore, compared the impact of HDM and *Alt* sensitization on *B. melitensis* infection in wild-type, IL-4^−/−^ and STAT6^−/−^ BALB/c mice. We observed that neutralization of the IL-4/STAT6 signaling pathway completely abrogates the enhanced susceptibility to *B. melitensis* infection due to both HDM and *Alt* sensitization (Figure 5), thus demonstrating that enhanced susceptibility to *Brucella* observed in asthmatic wild-type mice is strictly dependent on the Th2 response. Note that we have previously published (48) that the absence of STAT-6 signaling does not affect the course of *B. melitensis* infection in non-asthmatic BALB/c mice.

**Figure 6:**
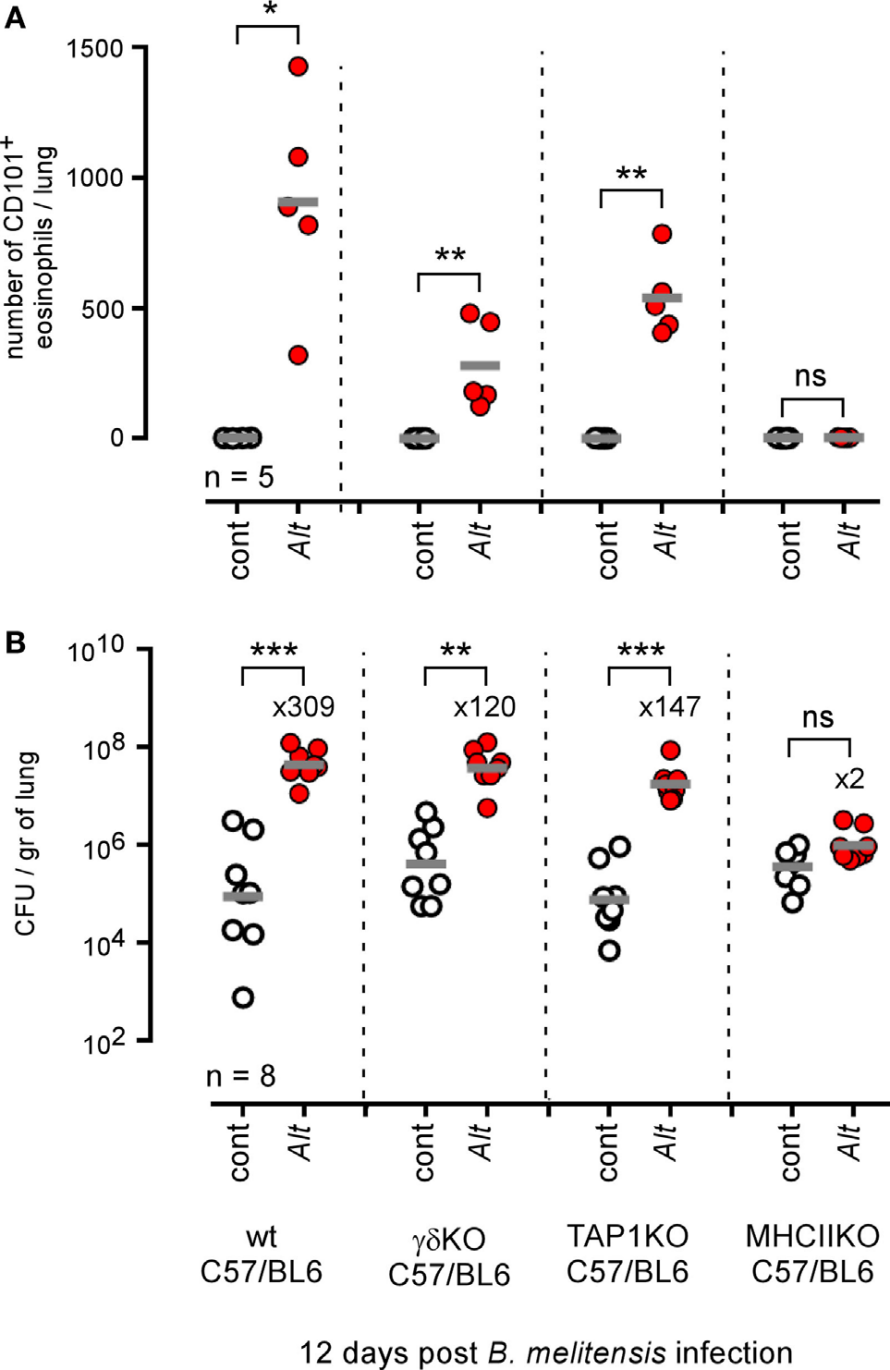
Impact of T lymphocyte deficiency on *Alt*-induced *Brucella* susceptibility. Wild-type, γδTCR^−/−^, TAP1^−/−^, and MHCII^−/−^ C57BL/6 mice received repeated i.n. administration of phosphate-buffered saline or *Alt* before i.n. infection with 2 × 10^4^ CFU of mCherry-*Brucella melitensis*. The mice were sacrificed at 12 days post infection. **(A)** The data represent the number of eosinophils recruited (SIGLEC-F^+^ CD11c^−^ CD101^+^) × 10^5^ per lung from each group as determined by flow cytometry. **(B)** The data represent the number of CFU/gr of lung at 12 days post infection from each group. Horizontal gray lines represent the medians. *n* denotes the number of mice used for each lineage. These results are representative of at least three independent experiments. *p < 0.05, **p < 0.01, ***p < 0.001.

As both αβ^+^CD4^+^ T cells (4) and γδ^+^ T cells (49) have been implicated in the development of allergic asthma in mice models, we next compared the ability of wild-type, γδ^−/−^, TAP1^−/−^ (CD8^+^ T cells deficient), and MHCII^−/−^ (CD4^+^ T cells deficient) C57BL/6 mice sensitized with *Alt* to control i.n. *B. melitensis* infection. Flow cytometry analysis (Figure 6A) showed that CD4^+^ T cell deficiency completely abrogated inflammatory eosinophil recruitment [SIGLEC-F^+^ CD11c^−^ CD101^+^ cells (50)] in infected asthmatic mice. The absence of γδ^+^ T cells and CD8^+^ T cells also significantly reduces eosinophil recruitment, though more moderately. However, we observed that only CD4^+^ T cell deficiency impaired *Alt*-induced susceptibility to *Brucella* infection significantly (Figure 6B).

### Allergic Asthma-Induced Susceptibility to *Brucella* Is not Associated With Preferential Multiplication in M2 Macrophages and Is Independent of Arginase-1 Activity

**Figure 7:**
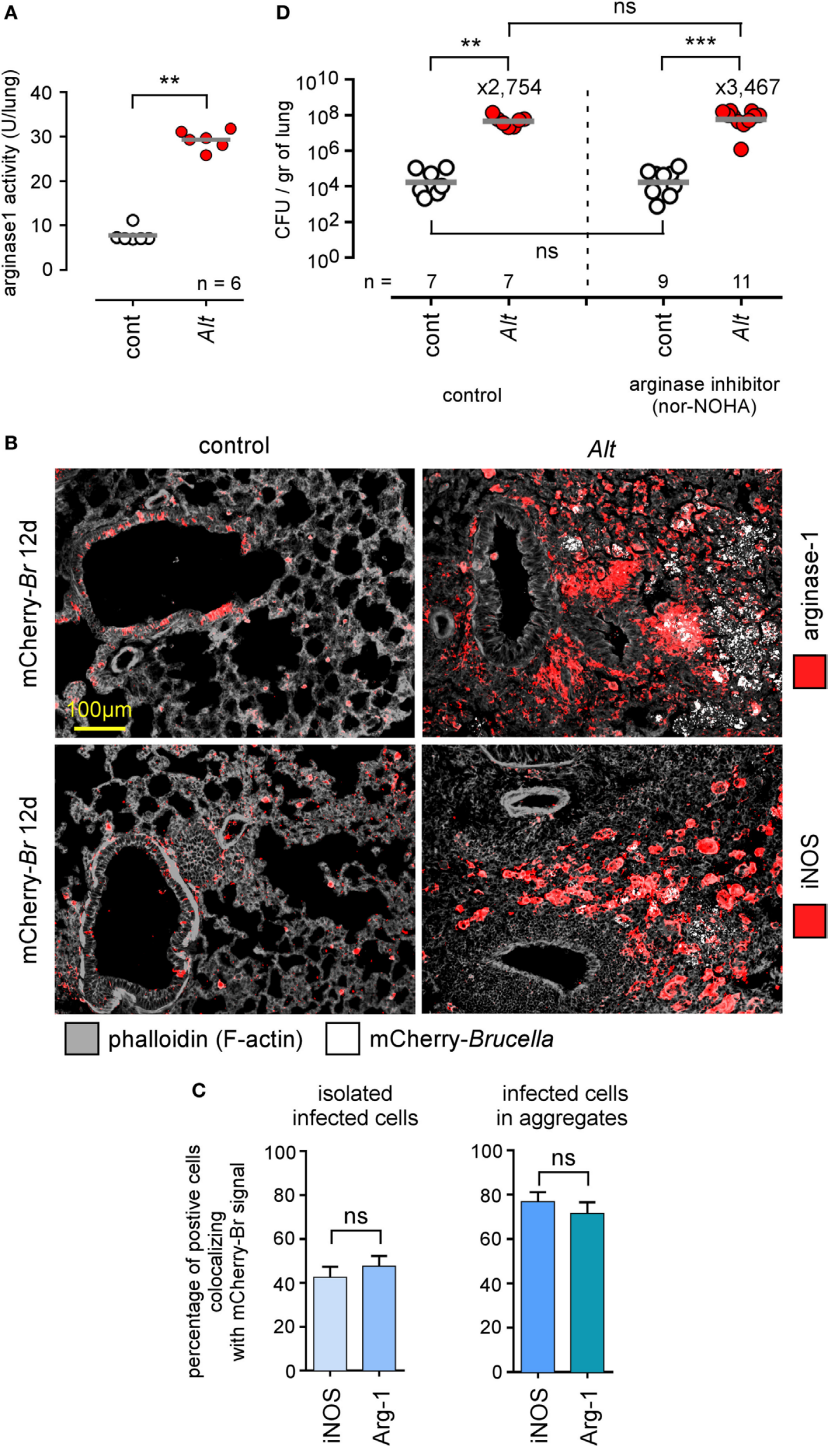
Asthma-induced *Brucella* susceptibility is independent of arginase activity. Wild-type BALB/c mice received repeated i.n. administration of phosphate-buffered saline (PBS) (control) or *Alt* before i.n. administration of PBS or 2 × 10^4^ CFU of mCherry-*Brucella melitensis*. The mice were sacrificed at 12 days post infection. The lungs were harvested and fixed. Frozen sections were examined by immunohistofluorescence for mCherry (*Brucella*), arginase-1 and iNOS signals. During the sensitization and infection process, the mice received repeated administration of PBS (control group) or the arginase inhibitor nor-NOHA. **(A)** The panel represents the arginase activity/lung homogenate from control and *Alt* sensitized wild-type BALB/c mice. **(B)** Representative view of mCherry^+^, arginase^+^, and iNOS^+^ cells in the lungs of control and *Alt* sensitized mice. The panels are color-coded by antigen as indicated. **(C)** The data represent the percentage of mCherry-*Br* signal that co-localizes with arginase-1 and iNOS staining. When >12 infected cells are observed in the same observation field (approximately 700 μm × 500 μm), they are considered as “aggregated,” and when <12 are observed, they are considered as “isolated.” At least 200 infected cells from three different infected mice were analyzed for each staining. **(D)** The panel represents the number of CFU/gr of lung at 12 days post infection from each group of mice. Horizontal gray lines represent the medians. *n* denotes the number of mice used for each lineage. These results are representative of at least two independent experiments. **p < 0.01, ***p < 0.001.

Allergic asthma is associated with increased polarization of alveolar macrophages toward an M2 phenotype characterized by high arginase-1 expression levels [reviewed in Ref. (51)]. It has been demonstrated that, *in vitro, B. abortus* multiply more actively in arginase-1^+^ M2 macrophages than in iNOS^+^ M1 macrophages (52), and we have reported that *B. melitensis* splenic reservoir cells from infected IL-12^−/−^ mice express high levels of arginase-1 (48). Thus, we tried to determine whether asthma-induced susceptibility to *Brucella* infection could be the consequence of the preferential invasion by *Brucella* of more abundant M2 macrophages present in asthmatic lungs. As expected, *Alt* sensitization significantly increases arginase activity in the lungs (Figure 7A) and microscopic analysis showed that infected asthmatic mice display an increase number of arginase-1^+^ cells (Figure 7B). However, these mice also display much higher numbers of iNOS^+^ cells (Figure 7B) compared to infected control mice. Microscopic analysis showed that infected cells colocalize with arginase-1 and iNOS staining at an almost equivalent frequency (Figure 7C), demonstrating that *Brucella* multiplication is no more associated with the M1 profile than with the M2 profile in our experimental model. We also observed that neutralization of arginase activity by repeated administration of nor-NOHA, a specific arginase inhibitor known to have the capacity to attenuate allergic airway inflammation (53) and neutralize arginase activity of asthmatic lungs *in vitro* (Figure S3 in Supplementary Material), does not reduce susceptibility to *Brucella* infection induced by *Alt*-sensitization (Figure 7D). On the whole, these data suggest that asthma-induced susceptibility to *Brucella* is not dependent on preferential invasion of M2 macrophages or arginase activity.

### Allergic Asthma-Induced Susceptibility to *Brucella* Is Partially Dependent on IL-10 but Independent of IL-12

**Figure 8:**
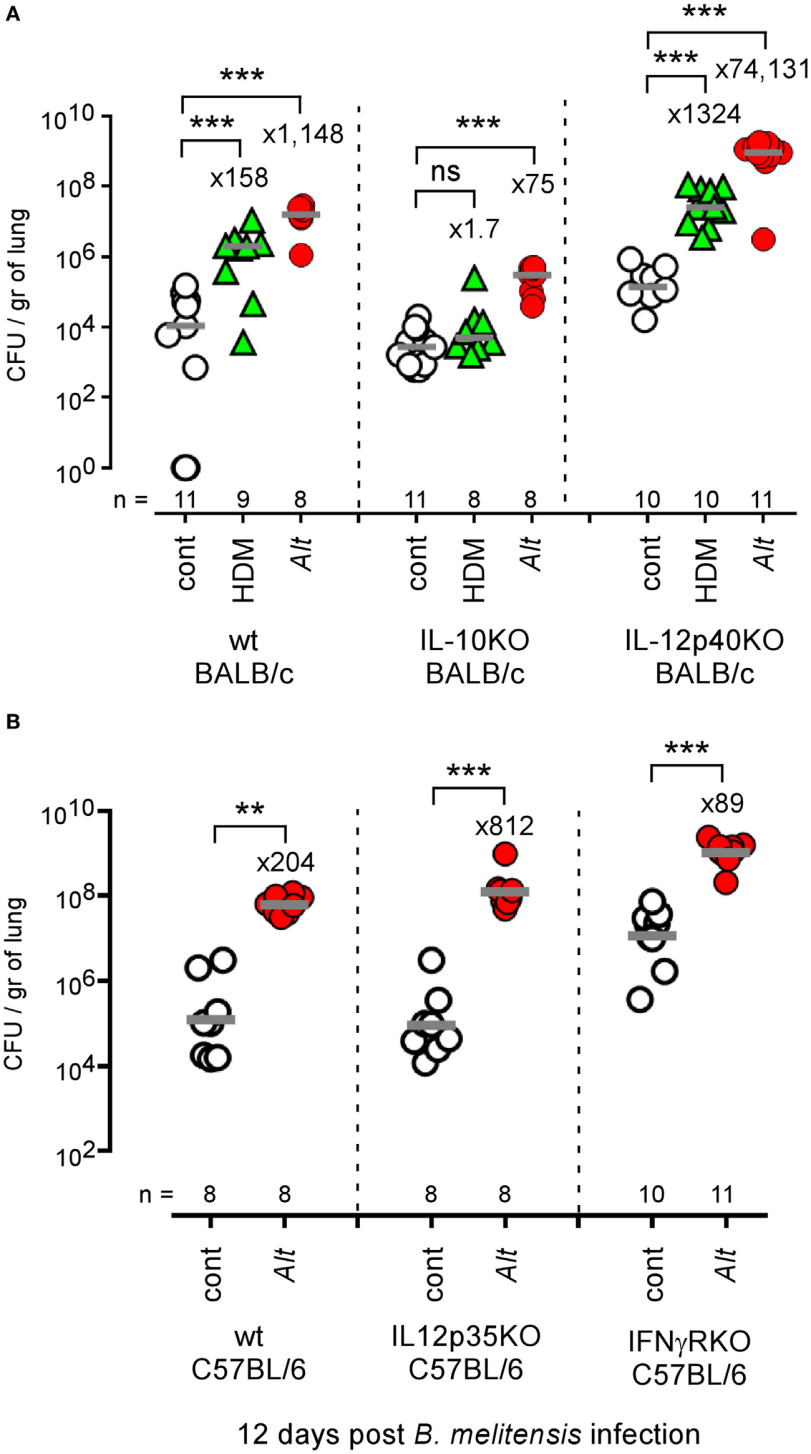
Impact of IL-10 and IL-12 deficiency on asthma-induced *Brucella* susceptibility. **(A)** Wild-type, IL-10^−/−^, and IL-12p40^−/−^ BALB/c mice received repeated i.n. administration of phosphate-buffered saline (PBS) or *Alt* before i.n. infection with 2 × 10^4^ CFU of mCherry-*Brucella melitensis*. The mice were sacrificed at 12 days post infection. The data represent the CFUs per gram of lung. **(B)** Wild-type, IL-12p35^−/−^, and IFN-γR^−/−^ C57BL/6 mice received repeated i.n. administration of PBS or *Alt* before i.n. infection with 2 × 10^4^ CFU of mCherry-*B. melitensis*. The mice were sacrificed at 12 days post infection. The data represent the CFU/gr of lung. Horizontal gray lines represent the medians. *n* denotes the number of mice used for each lineage. These results are representative of at least three independent experiments. **p < 0.01, ***p < 0.001.

**Figure 9:**
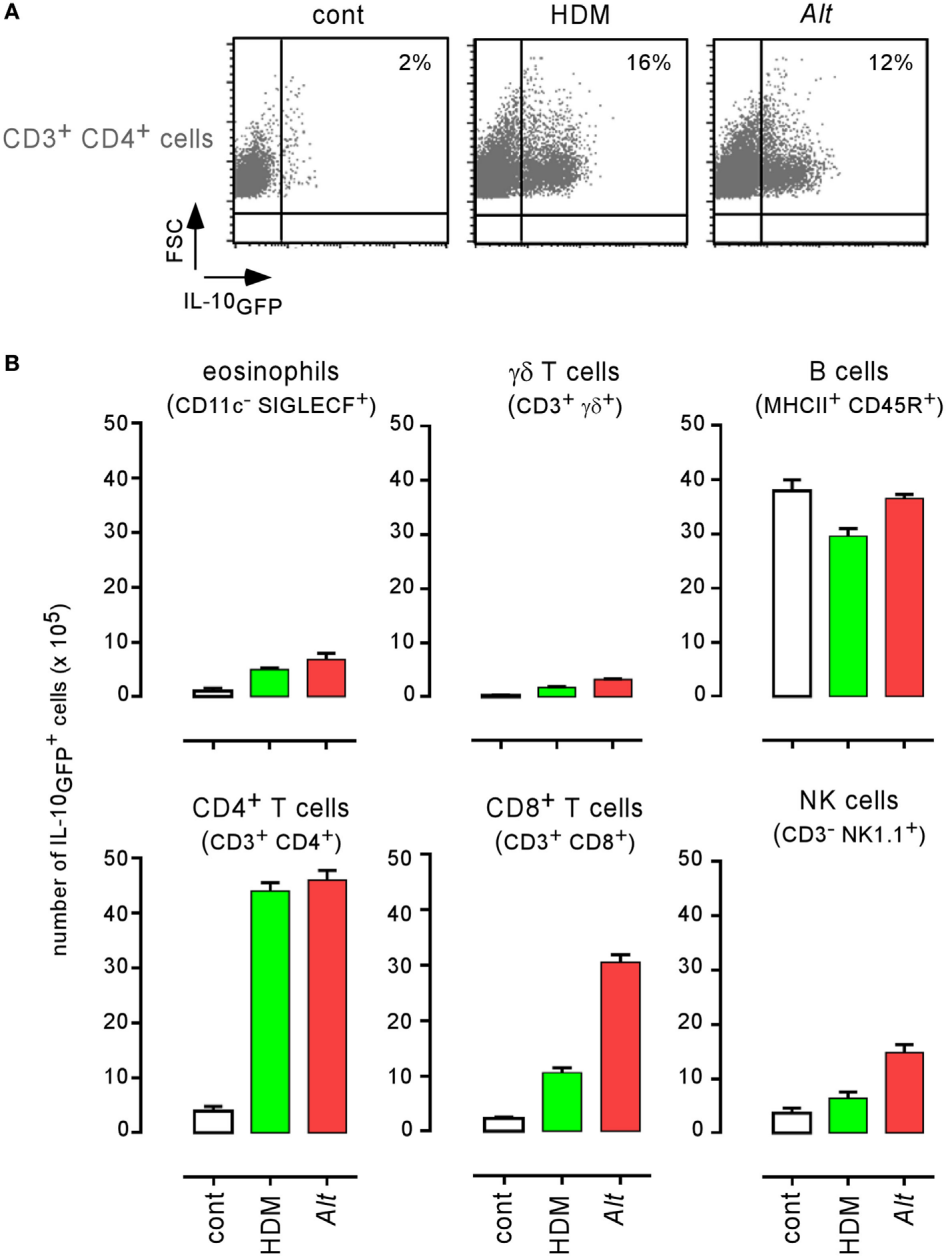
Characterization of IL-10 producing cells in lungs from infected asthmatic mice. IL-10_GFP_ transgenic C57BL/6 mice were sensitized with phosphate-buffered saline (PBS) (control group), HDM, or *Alt* before i.n. infection with 2 × 10^4^ CFU of *Brucella melitensis*. The mice were sacrificed at 12 days post infection, and the spleen cells were analyzed by flow cytometry. **(A)** Cells from PBS, HDM, and *Alt* infected mice were analyzed for IL-10GFP, CD3, and CD4 expression. The figure shows representative dot plots from individual lungs in each group, as indicated in the figure. Numbers represent the percentage of IL-10GFP^+^ cells in 50,000 acquired events. **(B)** The data represent the number of IL-10GFP^+^ eosinophils, γδ^+^ T cells, B cells, CD4^+^ T cells, CD8^+^ T cells, and natural killer cells per 10^5^ lung cells from control, HDM, and *Alt* infected mice. The data are representative of two independent experiments.

IL-10 is an anti-inflammatory cytokine notably produced by T cells (54) and B cells (55) during the asthma reaction. It is a master negative regulator of the Th1 response (56, 57) able to reduce the protective immune response against *Brucella* (58). We compared the ability of HDM and *Alt* sensitized wild-type, IL-10^−/−^, and IL-12p40^−/−^ BALB/c mice to control *B. melitensis* infection. While IL-10 deficiency in BALB/c mice strongly reduces asthma-induced susceptibility (158- to 1.7-fold increase for HDM and 1,148- to 75-fold increase for *Alt*), this is not the case of IL-12p40 deficiency (158- to 1,324-fold increase for HDM and 1,148- to 74,131-fold increase for *Alt*) (Figure 8A). A similar result was observed in C57BL/6 mice, thus demonstrating that this result is not dependent on the mouse strain used. The absence of IL12p35 and IFN-γR in C57BL/6 mice does not reduce (204- to 812-fold increase) or affects weakly (204- to 89-fold increase) the impact of asthma sensitization on *Brucella* levels, respectively (Figure 8B). Flow cytometry analysis of lung cells from control, HDM, and *Alt* sensitized infected transgenic mice expressing GFP under the control of the IL-10 promoter (IL-10_GFP_ mice) showed that IL-10 is mainly produced by B cells in control mice and by B cells, CD4^+^ T cells, and to a lesser extent eosinophils, CD8^+^ T and NK1.1^+^ cells following asthma sensitization (Figure 9). Unfortunately, alveolar macrophages display high levels of autofluorescence in the GFP channel (59), thus rendering analysis of their IL-10 expression in IL-10_GFP_ mice impossible. Taken together, these results suggest that IL-10 is a major mediator of HDM and *Alt*-induced susceptibility to *B. melitensis* lung infection, but that this phenomenon is independent of neutralization of the IL-12-dependent Th1 signaling pathway.

### Allergic Asthma Does not Affect Protective Immune Memory Against *Brucella*

**Figure 10:**
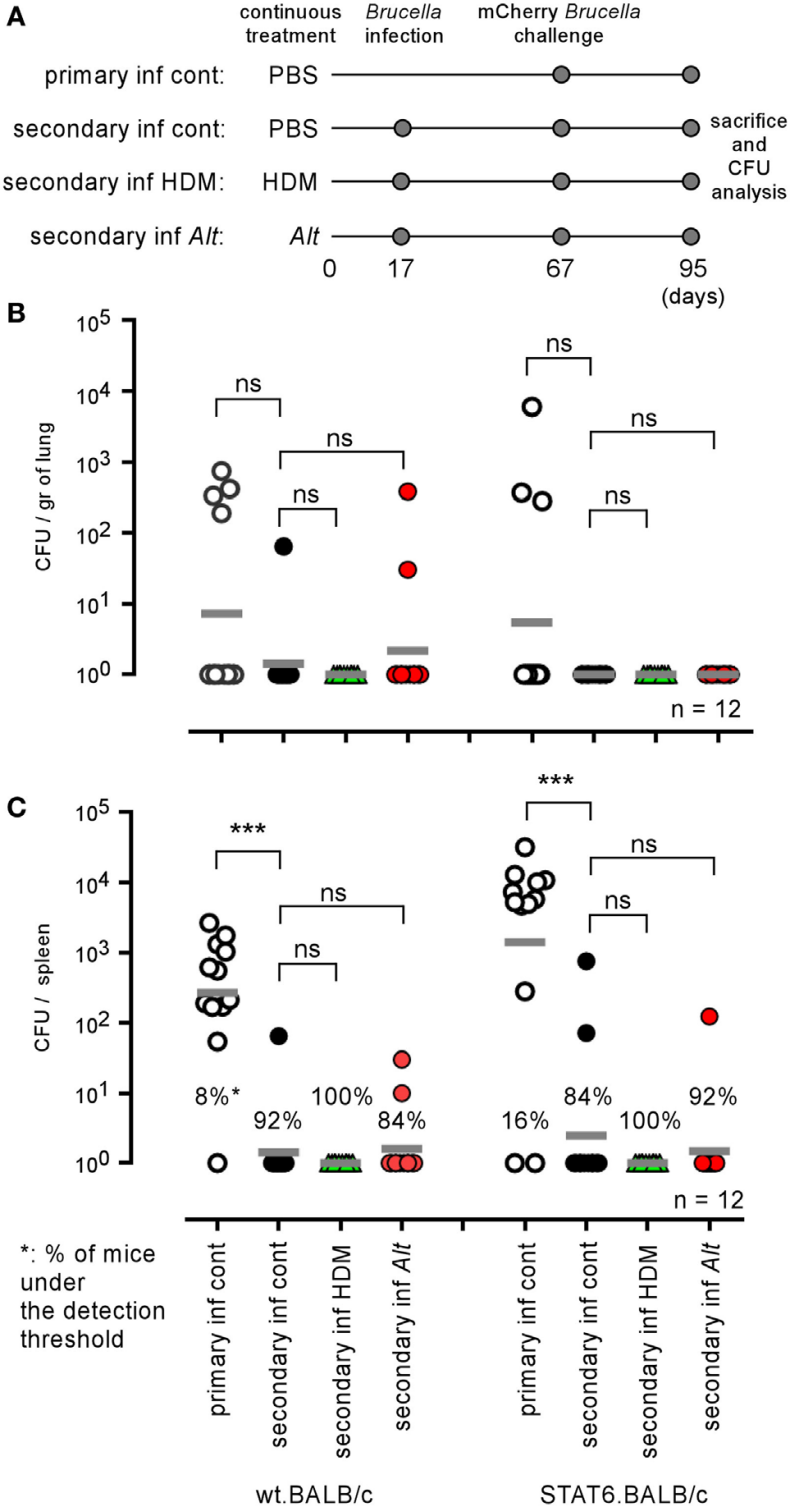
Influence of allergic asthma on the development of protective memory against *Brucella melitensis*. Wild-type and STAT-6^−/−^ BALB/c mice were sensitized with phosphate-buffered saline (PBS), HDM, or *Alt* extracts, as described in the Section “Materials and Methods,” throughout the entire experiment. Primary and secondary groups received i.n. administration of PBS or 2 × 10^4^ CFU of wild-type *B. melitensis* at 17 days, respectively. All groups were infected i.n. with 2 × 10^4^ CFU of mCherry-*B. melitensis* at 67 days, were sacrificed at 95 days, and the spleens were harvested and the CFUs were counted. **(A)** Is a schematic representation of the protocol. **(B,C)** Represent the number of CFU/gr of lung **(B)** and spleen **(C)**. Horizontal gray lines represent the medians. *n* denotes the number of mice used for each lineage. These results are representative of at least two independent experiments. ***p < 0.001.

As both HDM and *Alt* sensitization strongly favor the growth of *B. melitensis* in the lungs, we tried to determine whether they also affect the development of protective memory. We compared the ability of groups of wild-type and STAT-6^−/−^ BALB/c mice sensitized with PBS, HDM, or *Alt* during the course of a primary i.n. *Brucella* infection to control a secondary i.n. infection. As described in Figure 10A, mice were sensitized for 17 days with PBS (control), HDM, or *Alt*. before receiving 2 × 10^4^ CFU of wild-type *B. melitensis* i.n. Fifty days later, the mice were challenged with mCherry-expressing *B. melitensis* and sacrificed 28 days post challenge. Note that the sensitization treatment was continued during both primary infection and secondary infection in order to mimic chronic asthma inflammation. As illustrated in Figures 10B,C, we observed that all groups of wild-type and STAT-6^−/−^ BALB/c mice, whether sensitized or not, displayed a similar ability to control secondary *Brucella* infection in the spleen or lungs, suggesting that chronic asthma does not significantly affect the development of protective memory against i.n. *B. melitensis* infection.

### Allergic Asthma Increases Resistance to *S. pneumoniae* Infection in Mice

As the *Alt* sensitization protocol strongly increases the susceptibility of mice to pulmonary *Brucella* infection, we tried to determine whether this effect could be generalized to other lung infections with extracellular or intracellular bacteria. Therefore, we analyzed the impact of *Alt* sensitization on *S. pneumoniae* and *M. tuberculosis* infection in mice, two common serious human pulmonary pathogens.

**Figure 11:**
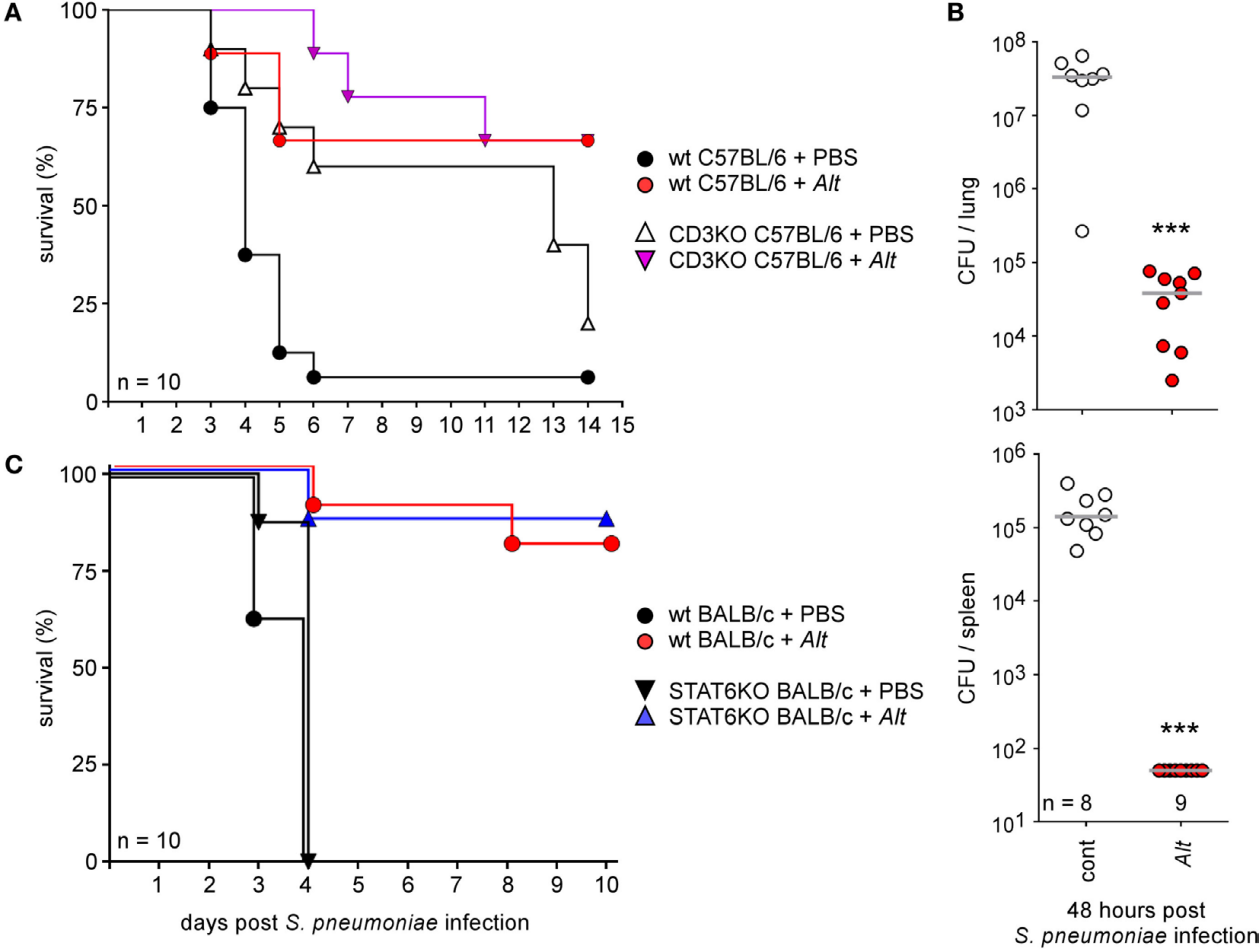
Asthma sensitization increases resistance against intranasal *Streptococcus pneumoniae* infection. Wild-type and CD3^−/−^ C57BL/6 **(A,B)** and wild-type and STAT-6^−/−^ BALB/c mice **(C)** were sensitized with phosphate-buffered saline (cont) or *Alt* before i.n. infection with a lethal CFU dose (2 × 10^7^ CFU) of *S. pneumonia*, as indicated in the legend of each graph. **(A,C)** The data shown are the percentage of mice surviving infection at a given time. **(B)** The data represent the number of CFU per lung or spleen, as indicated. *n* indicates the number of mice per group. These results are representative of at least two independent experiments.

Control and *Alt* sensitized wild-type C57BL/6 (Figures 11A,B) and BALB/c (Figure 11C) mice were infected i.n. with 2 × 10^7^ CFU of *S. pneumoniae*. This bacterial dose is 95–100% lethal in control C57BL/6 and BALB/c mice, but appears to be well tolerated by asthmatic mice. Only 10–15% of the latter died after 10 days of infection. As expected, asthma-induced resistance is associated to a drastic reduction of CFU count in lung and spleen 48 h post infection (Figure 11B). Surprisingly, asthma-induced resistance to *S. pneumoniae* is also observed in sensitized CD3^−/−^ C57BL/6 mice (Figure 11A) and STAT-6^−/−^ BALB/c mice (Figure 11C), demonstrating that the protective effect of asthma is independent of Th2 CD4^+^ T cells.

**Figure 12:**
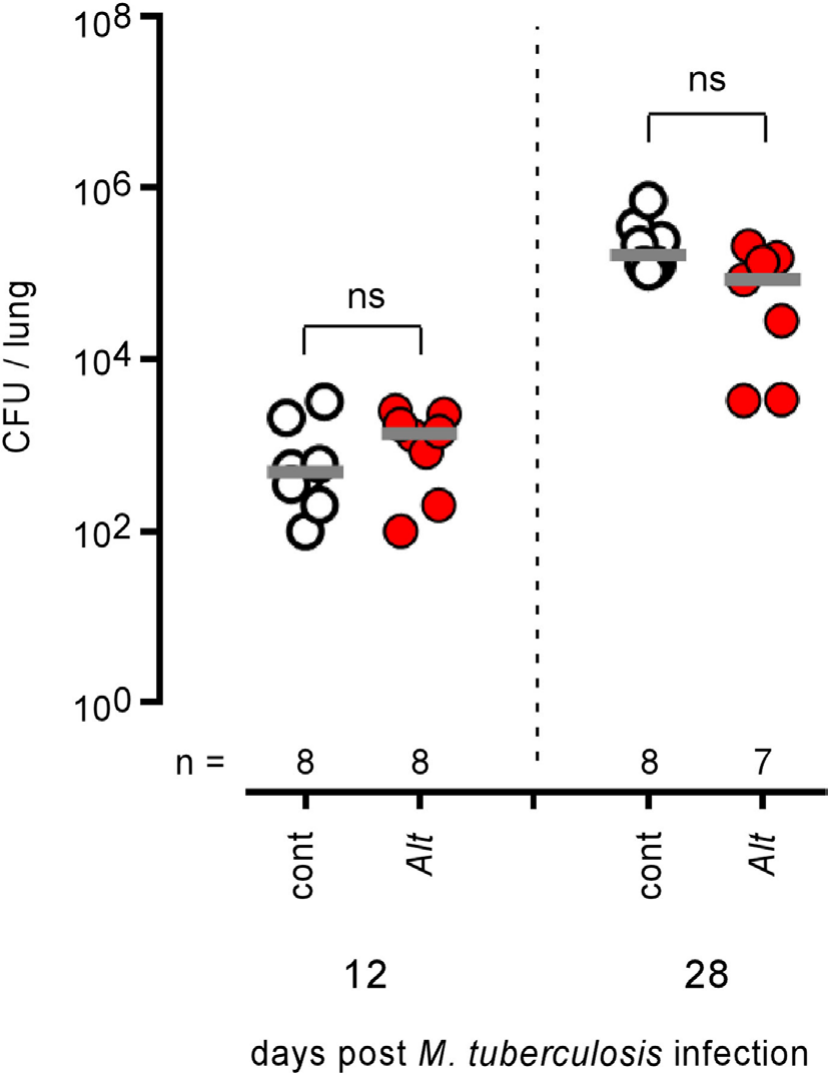
Asthma sensitization does not affect the course of *Mycobacterium tuberculosis* infection in the lungs of mice. Wild-type BALB/c mice were sensitized with phosphate-buffered saline (cont) or *Alt* before aerosol infection with 10^2^ CFU of *M. tuberculosis*. Mice were sacrificed at the indicated time post *M. tuberculosis* infection. The data show the CFU/lung. *n* indicates the number of mice per group. These results are representative of at least two independent experiments.

Control and *Alt* sensitized wild-type BALB/c mice (Figure 12) were also infected by aerosol with 100 CFU of *M. tuberculosis*. We observed that control and asthmatic mice display similar CFU counts in the lungs at 12 and 28 days post infection, suggesting that *Alt* sensitization does not significantly affect the course of *M. tuberculosis* infection in the lungs.

On the whole, these results demonstrate that the same asthma sensitization protocol can affect the ability of the immune system to control lung bacterial infections in very different and unpredictable ways.

## Discussion

Allergic asthma is one of the most common chronic inflammatory lung diseases affecting humans (2, 3). The risk factors for developing asthma are a combination of genetic predisposition along with environmental exposure to inhaled substances. The most common indoor allergens are derived from dust mites, mammals (including wild rodents and pets), and fungi (60). Infection also plays a well-documented role in the development of asthma (5). Conversely, the impact of asthma as a predisposing or aggravating condition to infection has been documented [for a review, see Ref. (61)] but has rarely been clearly addressed experimentally. Allergic asthma is dominated by the IL-4 (Th2) immune response that is well known since the pioneering work of Mossman and Coffman (35) to counter-regulate the IFN-γ dominated (Th1) immune response that controls viral and bacterial infection. For example, the chronic Th2 response induced by helminth infections has been associated in numerous experimental models with an impaired Th1 response to intracellular (36–38) and extracellular (39) bacteria as well as viruses (40, 41) and can reduce the efficacy of vaccines (62–64). However, contradictory results have been also reported, showing no effect (65) or even boosting effect (66) of helminth infections on the Th1 response.

In the present study, we analyzed the impact of chronic i.n. sensitization with house dust mite *D. farinae* (HDM) or mold *A. alternata* (*Alt*) extracts on the course of *Brucella* spp*., S. pneumoniae*, and *M. tuberculosis* lung infections. We observed that both asthma sensitization protocols strongly enhanced the growth of *Brucella melitensis* in the lungs, but not in other organs such as the spleen or liver following i.n. infection. In particular, at 12 days post infection, *Alt* sensitization induced a 2 and 3 log CFU increase in the lungs of C57BL/6 and BALB/c mice, respectively. This increase is very surprising by comparison with the impact of IFNγ-R or αβTCR deficiency in C57BL/6 that only leads to a 1 to 1.5 log increase of *Brucella* in the lungs of C57BL/6 (23). The lungs of infected asthmatic mice display large aggregates of Ly-6G^+^ cells, presumably neutrophils, surrounding *Brucella* infected alveolar macrophages (CD11c^+^ F4/80^+^ cells). Infection is concentrated in these areas and large fractions of lung tissue seem uninfected and healthy. In keeping with this observation, mortality was not increased in the infected asthmatic mice, even after 100 days of chronic sensitization and infection (data not shown). Asthma sensitization also favors the growth of *B. abortus* and *B. suis* infection in the lungs, demonstrating that this phenomenon can be observed with the three main *Brucella* species described to infect humans.

Comparison of various mice strains displaying selective deficiencies for key elements of the immune response demonstrated that the impact of asthma on *Brucella* lung infection is strictly dependent on CD4^+^ T cells and IL-4/STAT6 pathways, which suggests that IL-4-producing Th2 CD4^+^ T cells are key actors of this phenomenon. IL-10 deficiency partially restores the control of *Brucella* multiplication in the lungs of asthmatic mice. Flow cytometry analysis of IL-10GFP transgenic mice showed that asthma sensitization increases the frequency of IL-10-producing cells in the lungs. Taken together, these data suggest that asthma-induced susceptibility is partially due to the inhibition by IL-10 of immune effector mechanisms controlling *Brucella* multiplication in the lungs. However, asthma-induced susceptibility is also observed in IL-12^−/−^ and IFN-γR^−/−^ mice, suggesting that asthma-induced IL-10 affects IL-12 independent effector mechanisms controlling *Brucella* in the lungs. As the non Th1 immune effectors controlling *Brucella* growth in the lungs remain largely unknown, we failed to identify the immune effectors suppressed by IL-10 in our model.

*Brucella abortus* has been reported to survive and replicate more efficiently in arginase-1^+^ M2 macrophages than in iNOS^+^ M1 macrophages *in vitro*, notably because M2 macrophages display increased intracellular glucose availability, which promotes *Brucella* replication (52). As higher M2 alveolar macrophage counts have been documented during allergic asthma (51), we tried to determine whether asthma-induced increased *Brucella* susceptibility could be the result of the preferential invasion of M2 macrophages by *Brucella*. Microscopic analysis showed a strong increase in the frequency of both iNOS^+^ and arginase-1^+^ cells in the lungs of *Alt* sensitized mice. However, we did not observe preferential co-localization of *Brucella* with iNOS or arginase-1 staining in the lungs of asthmatic mice. Moreover, arginase activity neutralization by repeated injection of nor-NOHA did not reduce *Brucella* susceptibility induced by *Alt* sensitization. These results suggest that the positive impact of asthma on *Brucella* multiplication in our experimental model was not dependent on invasion of the M2 macrophages. However, M2 macrophages have been described to actively suppress the Th1 response in several likely redundant ways such as *via* the chitinase-like 3 protein (Ym1) (67) and programmed death ligand 2 (PD-L2) (68). We, therefore, cannot exclude their implication in increased susceptibility to *Brucella* infection in asthmatic mice. We also cannot exclude that the increased multiplication of *Brucella* in the macrophages of asthmatic mice was not partially due to the fact that these cells are enriched in nutrients necessary for the growth of *Brucella*.

The extracellular, Gram-positive bacterium *S. pneumoniae* is a serious human pathogen that causes more than 50% of cases of community-acquired bacterial pneumonia and is the most common cause of death from infection in developed countries [for a review, see Ref. (69, 70)]. In mice models, innate immunity is crucial during the early phase of natural anti-pneumococcal host defenses, and alveolar macrophages and neutrophils play a key role in the control of bacteria. While clinical studies have identified asthma as a significant risk factor for invasive pneumococcal disease (7, 71–73), we observed that our *Alt* sensitization protocol dramatically increased the resistance of mice to *S. pneumoniae* infection, demonstrating that asthma-induced susceptibility to *Brucella* cannot be generalized to all bacterial infections in our experimental model and suggesting that asthma can also, in some cases, improve resistance to bacterial infection. Wildly conflicting results have been published on the impact of asthma on *S. pneumoniae* infection in mice. Intraperitoneally, OVA-sensitized mice have been reported to display reduced (74), similar (75), or enhanced (76, 77) susceptibility to infection. The discrepancies between these results may be connected with the serotype of *S. pneumoniae* used, the sanitary level of the mice, or a delay between infection and asthma challenge. Surprisingly, in our model, CD3 and STAT-6 deficiency did not impair resistance to *S. pneumoniae* infection following asthma sensitization, demonstrating that this phenomenon is induced by a route that is completely different from that of asthma-induced *Brucella* susceptibility. Stimulation of innate lung immunity with an aerosolized lysate of non-typeable *Haemophilus influenzae* confers a high level of protection against a challenge with otherwise lethal inocula of *S. pneumoniae* (78). Intranasal administration of flagellin from *Salmonella enterica serovar Typhimurium* protects from *S. pneumoniae* infection in wild-type and B- and T-cell-deficient SCID mice, but not in neutrophil-depleted mice (33). This suggests that strong neutrophilia induced by asthma sensitization in STAT-6^−/−^ mice (Figure S2 in Supplementary Material) could be sufficient to increase resistance to *S. pneumoniae* in our model. Interestingly, a recent study (79) in a mouse model reported that allergic asthma also decreases lung infection with *Klebsiella pneumoniae* in a neutrophil-dependent and IL-4- and IL-17-independent manner.

Finally, we also tested the impact of *Alt* sensitization on *M. tuberculosis* lung infection. Although the course of *M. tuberculosis* infection, like *Brucella* infection, is mainly controlled by IFN-γ-producing CD4^+^ T cells (80–82) in mice, we observed that asthma sensitization does not affect *M. tuberculosis* multiplication in the lungs. It is noteworthy that while different studies indicate that BCG vaccination or infection with *M. tuberculosis* is associated with a decreased risk of allergic diseases (83–85) and that active tuberculosis disease is associated with increased allergic sensitization (86) or protection against allergy (84), no clear association between asthma and increased susceptibility to *M. tuberculosis* infection has been reported to the best of our knowledge. Overall, this result indicates again that the impact of *Alt* sensitization on *Brucella* infection cannot be generalized to all other intracellular bacterial infections.

We observed recently (87) that the strong Th1 response induced by *Trypanosoma brucei* infection significantly reduced *Brucella* growth but did not affect the course of *M. tuberculosis* infection in mice. Taken together, the absence of an impact of the Th1 response induced by *T. brucei* and the Th2 response induced by asthmatic sensitization on *M. tuberculosis* infection in our mice models is very surprising and suggests that control of *M. tuberculosis* is not simply dependent on a balance between the Th1 and Th2 responses.

On the whole, our results demonstrated that the same protocol for allergic asthma sensitization might have no effect or dramatically reduce or increase resistance to bacterial infection in mice, depending on the infectious agent. These results open up new areas for investigation of immune effectors controlling *Brucella* and *S. pneumoniae* growth in the lungs, and suggest that it could be interesting to perform further clinical studies on the impact of asthma on *Brucella* and *S. pneumoniae* infections in humans.

## Ethics Statement

The procedures used in this study and the handling of the mice complied with current European legislation (directive 86/609/EEC) and the corresponding Belgian law “Arrêté royal relatifà la protection des animaux d’expérience du 6 avril 2010 publié le 14 mai 2010.” The Animal Welfare Committee of the Université de Namur (UNamur, Belgium) reviewed and approved the complete protocol for Brucella infections (Permit Number: UN-LE-14/220). The Animal Welfare Committee of the Université Libre de Bruxelles (ULB, Belgium) reviewed and approved the complete protocol for *Streptococcus pneumoniae* infections (Permit Number: ULB-IBMM-2016-21-88). The Ethics committee of the WIV-ISP and CODA-CERVA approved the complete protocol for *Mycobacterium tuberculosis* infections (ethics agreement number 201405-14-01).

## Author Contributions

All authors listed have made a substantial, direct, and intellectual contribution to the work and approved it for publication.

## Conflict of Interest Statement

The authors declare that the research was conducted in the absence of any commercial or financial relationships that could be construed as a potential conflict of interest.

## Supplementary Material

Figure S1 Comparison of allergic asthma models induced by intranasal sensitization with HDM or *Alt* extracts. Wild-type BALB/c mice were instilled i.n. with phosphate-buffered saline (control), HDM, or *Alt* extracts for 17 days to induce allergic asthma. Three days after the last sensitization, the mice were sacrificed and the lungs were collected to evaluate the asthma severity. **(A)** Hematoxylin and eosin (HE) staining of lung paraffin sections (scale bar: 200 µm). **(B)** Differential cell counts in bronchoalveolar lavages fluid. **(C)** Q-PCR analysis of IL-4 mRNA expression levels in the lungs. **(D)** Total blood IgE Ab concentration determined by ELISA. *n* denotes the number of mice used for each lineage. These results are representative of at least two independent experiments. **p* < 0.05, ***p* < 0.01.

Figure S2 Impact of IL-4 and STAT6 deficiency on asthma-induced cell recruitment in the lungs of BALB/c mice. Wild-type, IL4^−/−^ and STAT6^−/−^ BALB/c mice received repeated i.n. administration of phosphate-buffered saline, HDM, or *Alt* before i.n. infection with 2 × 10^4^ CFU of mCherry-*Brucella melitensis*. The mice were sacrificed at 12 days post infection to evaluate the severity of the allergic phenotype. **(A)** Circulating blood non-specific IgE Ab concentration determined by ELISA. **(B)** Number of cell counts in bronchoalveolar lavage fluid from control, HDM, and *Alt* infected mice. These results are representative of at least two independent experiments. ***p* < 0.01, ****p* < 0.001.

Figure S3 Treatment with nor-NOHA inhibitor neutralizes arginase activity in lung homogenates from *Alt* sensitized BALB/c mice. Wild-type BALB/c mice received repeated i.n. administration of phosphate-buffered saline (PBS) (control) or *Alt* for 2 weeks. The mice were sacrificed and the lungs were harvested and homogenized. The panel represents the arginase activity/lung homogenate from control and *Alt* sensitized wild-type BALB/c mice incubated *in vitro* with PBS or nor-NONA inhibitor (2 mg/ml). These results are representative of at least two independent experiments. ***p* < 0.01, ****p* < 0.001.
