# Supplementary figures and images for: Allergic Asthma Favors Brucella Growth in the Lungs of Infected Mice
Source: Front Immunol. 2018 Aug 10;9:1856. doi: 10.3389/fimmu.2018.01856 (PMC6095999; doi:10.3389/fimmu.2018.01856)

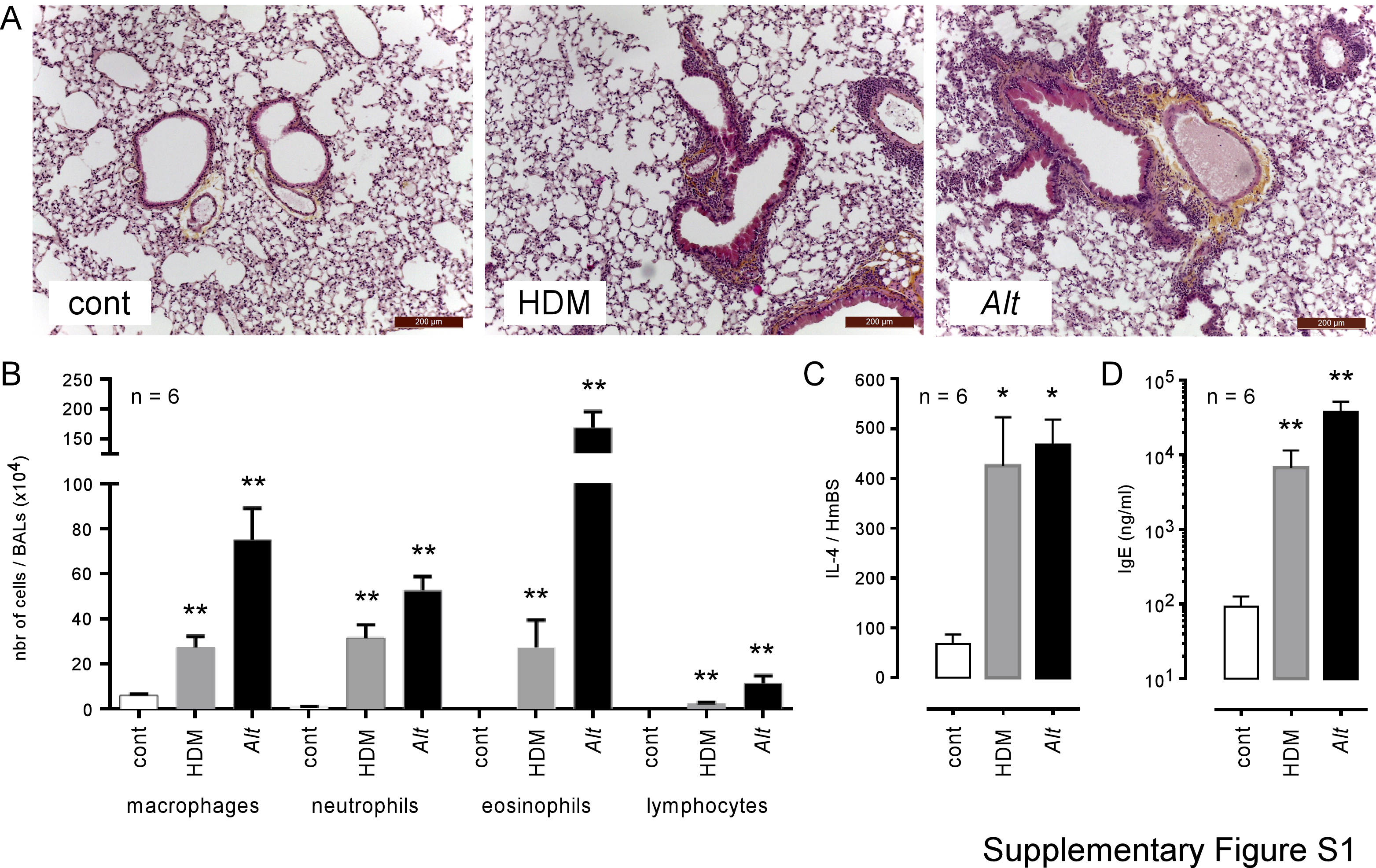

Supplement: Figure S1 — Comparison of allergic asthma models induced by intranasal sensitization with HDM or Alt extracts. Wild-type BALB/c mice were instilled i.n. with phosphate-buffered saline (control), HDM, or Alt extracts for 17 days to induce allergic asthma. Three days after the last sensitization, the mice were sacrificed and the lungs were collected to evaluate the asthma severity. (A) Hematoxylin and eosin (HE) staining of lung paraffin sections (scale bar: 200 µm). (B) Differential cell counts in bronchoalveolar lavages fluid. (C) Q-PCR analysis of IL-4 mRNA expression levels in the lungs. (D) Total blood IgE Ab concentration determined by ELISA. n denotes the number of mice used for each lineage. These results are representative of at least two independent experiments. *p < 0.05, **p < 0.01. [file image_1.tif]

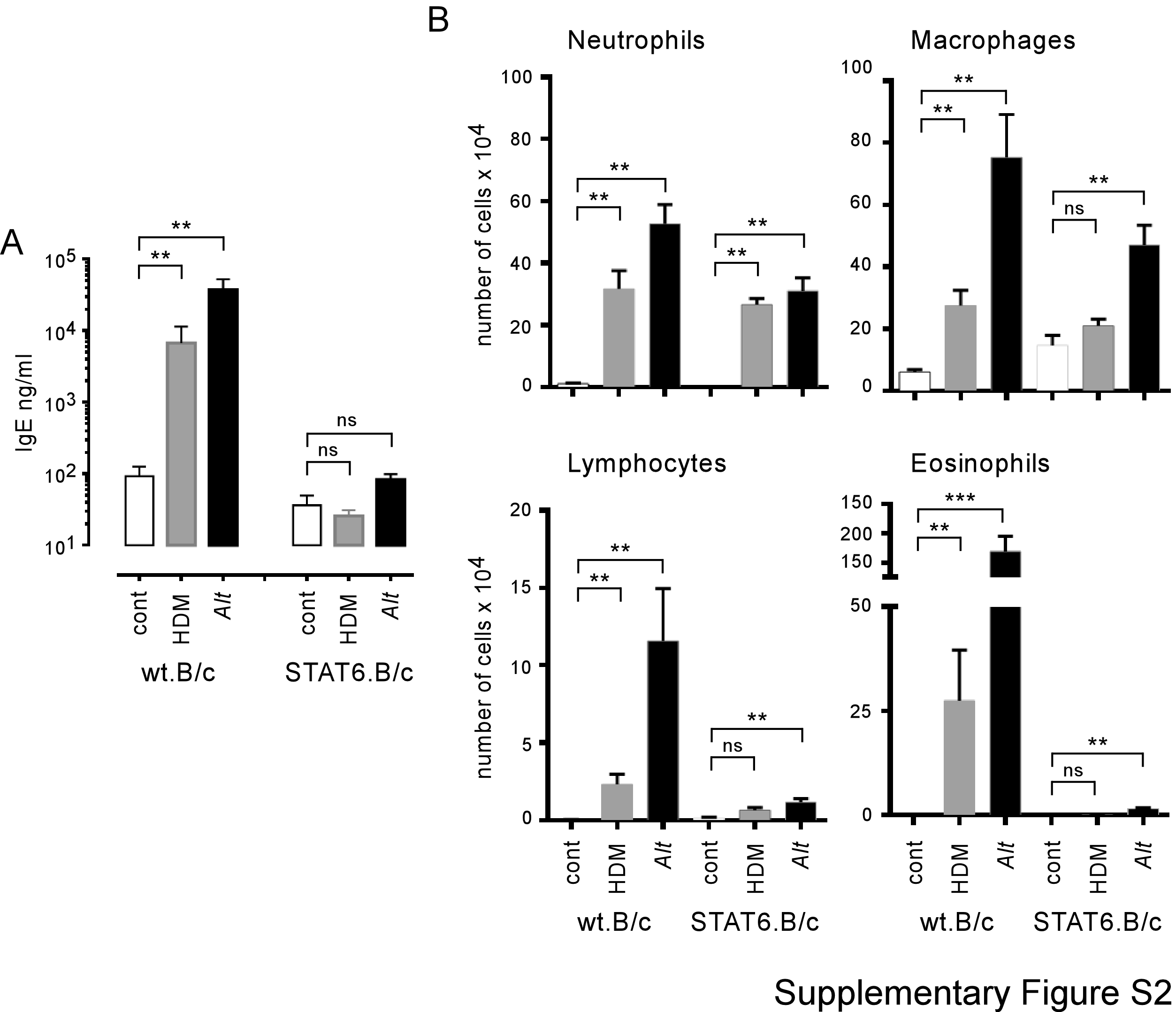

Supplement: Figure S2 — Impact of IL-4 and STAT6 deficiency on asthma-induced cell recruitment in the lungs of BALB/c mice. Wild-type, IL4−/− and STAT6−/− BALB/c mice received repeated i.n. administration of phosphate-buffered saline, HDM, or Alt before i.n. infection with 2 × 10^4^ CFU of mCherry-Brucella melitensis. The mice were sacrificed at 12 days post infection to evaluate the severity of the allergic phenotype. (A) Circulating blood non-specific IgE Ab concentration determined by ELISA. (B) Number of cell counts in bronchoalveolar lavage fluid from control, HDM, and Alt infected mice. These results are representative of at least two independent experiments. **p < 0.01, ***p < 0.001. [file image_2.tif]

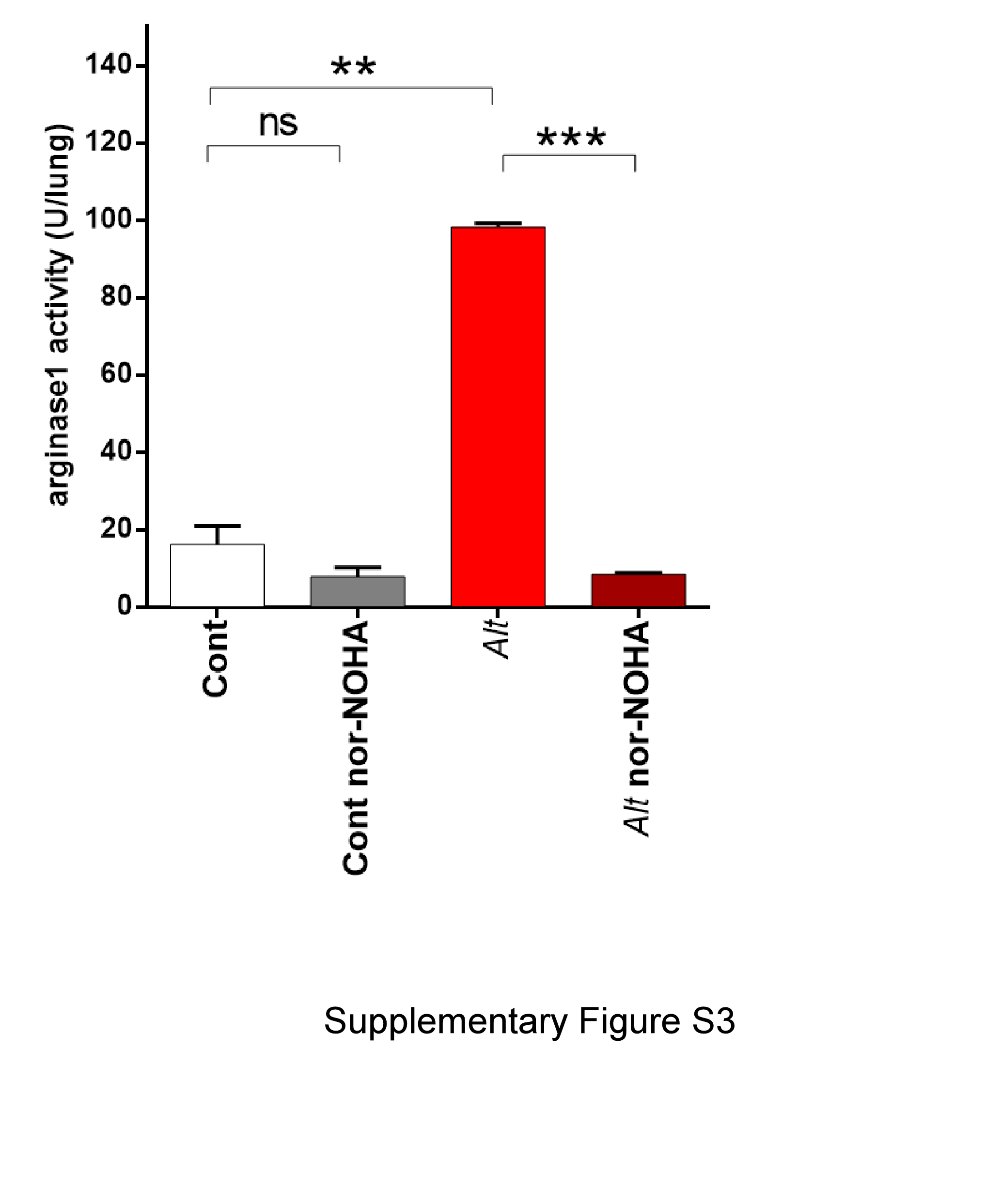

Supplement: Figure S3 — Treatment with nor-NOHA inhibitor neutralizes arginase activity in lung homogenates from Alt sensitized BALB/c mice. Wild-type BALB/c mice received repeated i.n. administration of phosphate-buffered saline (PBS) (control) or Alt for 2 weeks. The mice were sacrificed and the lungs were harvested and homogenized. The panel represents the arginase activity/lung homogenate from control and Alt sensitized wild-type BALB/c mice incubated in vitro with PBS or nor-NONA inhibitor (2 mg/ml). These results are representative of at least two independent experiments. **p < 0.01, ***p < 0.001. [file image_3.tif]
